# Assessment of biochar filter application in improving chromium stress tolerance and plant physiology in Chinese cabbage (*Brassica rapa*) under a flow-through water setup

**DOI:** 10.1186/s12896-025-01010-3

**Published:** 2025-07-19

**Authors:** Shuangqi Yue, Weidong Li, Fengyue Qin, Menglu Dong, Guojie Weng, Hayssam M. Ali, Jiechang Weng, Sajid Mehmood

**Affiliations:** 1 Center for Eco-Environment Restoration of Hainan Province, School of Ecology, Hainan University, Haikou, 570228 China; 2 School of Topical Agriculture and Foresty, Hainan University, Haikou, 570228 China; 3 Department of Botany and Microbiology, College of Science, King Saud University, Riyadh, 11451 Saudi Arabia; 4 Hainan Provincial Ecological and Environmental Monitoring Center, Haikou, 570228 China

**Keywords:** *Brassica rapa* L., Chromium stress, Biochar filtration, Oxidative stress mitigation, Hydroponic agriculture

## Abstract

**Background:**

With the increasing use of industrial wastewater for irrigation and the growing prevalence of heavy metal contamination in soils, chromium (Cr) pollution poses a significant threat to crop safety, particularly in industrially concentrated regions. Although biochar has been widely applied in soil remediation, its potential use as a real-time filtration medium in dynamic hydroponic systems remains largely unexplored. To address this gap, the present study investigated the efficacy of different biochar concentrations (0.5, 1.25, and 2.5 g/L) in mitigating Cr-induced stress (20 mg/L Cr (VI)) in a hydroponic system using *Brassica rap*a L., a fast-growing, Cr-sensitive leafy vegetable, as a model crop. The study aimed to evaluate plant growth and physiological responses under Cr stress and provide innovative strategies for protected agriculture.

**Results:**

Scanning Electron Microscopy (SEM) and Brunauer-Emmett-Teller (BET) analysis revealed a highly porous biochar structure, while Fourier Transform Infrared Spectroscopy (FTIR) confirmed the presence of key functional groups (–OH,–COOH) essential for Cr adsorption. X-ray Diffraction (XRD) indicated the presence of well-crystallized minerals such as quartz. Additionally, X-ray Photoelectron Spectroscopy (XPS) analysis verified the successful adsorption of chromium on the biochar surface and revealed the coexistence of Cr (VI) and Cr (III) species, indicating that partial reduction of Cr (VI) occurred during the adsorption process-likely facilitated by redox-active oxygen-containing groups. In the absence of biochar, Cr exposure significantly reduced plant biomass, chlorophyll content, and antioxidant enzyme activity while increasing oxidative stress markers and Cr accumulation in plant tissues. In contrast, biochar treatments-particularly at 2.5 g/L-significantly improved plant growth, enhanced chlorophyll and antioxidant activity, decreased Cr accumulation in roots and shoots, and alleviated oxidative stress. At this optimal dose, soluble sugar and protein contents increased by 52.8% and 114.4%, respectively. Correlation analysis showed a strong negative relationship between Cr accumulation and growth traits, and a positive correlation between antioxidant enzyme activity and stress mitigation. Hierarchical Cluster Analysis (HCA) and radar chart visualizations further confirmed the distinct physiological profiles induced by biochar, with 2.5 g/L treatment demonstrating the most balanced improvements across multiple traits.

**Conclusions:**

This study is the first to explore the use of biochar as a dynamic filtration medium for Cr remediation in hydroponic systems, demonstrating its dual role in Cr adsorption and physiological stress alleviation. The 2.5 g/L dose was identified as optimal, reducing Cr accumulation in aerial tissues by 62.4% and increasing soluble protein content by 114%. These findings offer a practical and environmentally sustainable solution for managing heavy metal risks in hydroponic and urban agriculture, particularly in resource-limited settings. The proposed technology combines environmental and economic benefits, making it especially suitable for facility-based agricultural production systems.

**Supplementary Information:**

The online version contains supplementary material available at 10.1186/s12896-025-01010-3.

## Background

Rising global population, along with rapid industrialization and urbanization, has intensified environmental pollution. Among various pollutants, heavy metals in untreated industrial effluents pose serious threats to ecosystems and human health due to their persistence and toxicity [1]. Elements like lead, cadmium, mercury, arsenic, and chromium (Cr) are non-biodegradable and tend to accumulate in soils and sediments, creating long-term ecological risks [2, 3].

Chromium, widely used in electroplating, tanning, and dye manufacturing, enters the environment through industrial activities such as coal combustion, mining, and wastewater discharge, with over 30,000 tons released globally in the past five decades [4, 5]. Its accumulation in wastewater and soils poses increasing environmental and health concerns [6, 7]. Nationally, Cr contamination affects 11% of polluted sites in the U.S., 14% in Japan [8], and over 5% of total potentially toxic element-contaminated land in China [9]. Cr (VI) accumulation in agricultural soils disrupts crop productivity [1, 10] and contaminates surface and groundwater beyond safe limits, often exceeding regulatory thresholds [11]. Soil Cr levels typically range from 10 to 50 mg/kg [12], and toxicity thresholds for plants are 0.5-5 mg/L in nutrient solution and 5-100 mg/kg in soil [1]. The International Agency for Research on Cancer (IARC) classifies Cr (VI) as a human carcinogen [13]. Exposure levels as low as 0.1 mg/L can cause DNA damage and increase risks of lung, bladder, liver, and skin cancers, as well as respiratory and immune system disorders [14]. Dietary intake is considered the most common exposure pathway [15]. In agriculture, Cr pollution threatens global food security, affecting grain and vegetable production [16, 17]. Leafy vegetables like Chinese cabbage (*Brassica rapa*), a nutrient-rich crop with a short growth cycle, are especially vulnerable due to their high Cr uptake efficiency [18]. Cr exposure in *Brassica rapa* leads to growth inhibition, leaf morphological changes, and rhizosphere disruption [19, 20].

While several remediation strategies exist, many are unsuitable for flow-through systems or economically unviable. Conventional methods-chemical precipitation, ion exchange, and resin adsorption-are effective but limited by sludge generation, pH dependence, and secondary pollution [21, 22]. Given these limitations, there is a need for real-time, efficient, and eco-friendly remediation systems that can operate in hydroponic environments. Biochar, a porous, carbon-rich material produced by biomass pyrolysis, presents a promising alternative. Its high surface area, functional groups, and redox properties make it effective for heavy metal adsorption and immobilization [23–25]. Prior research highlights biochar’s role in reducing metal bioavailability, improving soil health, and enhancing plant resilience [26–28], but its use in hydroponic systems remains underexplored.

This study employs a flow-through hydroponic system to evaluate the efficacy of biochar filtration in mitigating Cr toxicity in *Brassica rapa*. We aim to (1) identify the optimal biochar dose (0.5, 1.25, or 2.5 g/L) for reducing Cr stress, and (2) assess biochar’s effects on growth, antioxidant activity, photosynthetic pigments, and elemental composition. This research builds on biochar’s established remediation potential to extend its application to controlled hydroponic environments, contributing to sustainable agricultural practices.

## Materials and methods

### Biochar synthesis and characterization

Biochar was prepared using the invasive plant *Ageratina adenophora* as the feedstock, selected for its availability and potential for valorization. Stems were cut into approximately 2.5 cm (1-inch) segments, thoroughly washed with deionized water to remove dust and impurities, and oven-dried at 60 °C for 24 h to eliminate moisture. The dried biomass was pulverized into a fine powder using a grinder and sieved through a 100-mesh screen to ensure uniform particle size, facilitating consistent pyrolysis [29]. The powdered biomass was then wrapped in aluminum foil, placed in a ceramic crucible, and pyrolyzed in a muffle furnace [30]. The temperature was increased at a rate of 10 °C per minute to a maximum of 250 °C, held for 2 h, and then allowed to cool naturally to room temperature. This slow pyrolysis method, carried out under oxygen-limited conditions, promotes the formation of a porous structure favorable for heavy metal adsorption, while the low operating temperature minimizes volatile emissions, thereby reducing environmental impact [26, 31, 32].

The surface morphology of the biochar was analyzed using scanning electron microscopy (SEM; Verios G4 UC, Thermo Fisher Scientific, Czech Republic), while elemental composition and spatial distribution were assessed through energy-dispersive spectrometry (EDS) on the same instrument. Functional groups were identified using Fourier-transform infrared spectroscopy (FTIR; Nicolet iS50, Thermo Scientific, USA), offering insights into surface chemistry relevant to heavy metal binding [33]. Phase composition and crystallinity were analyzed using X-ray diffraction (XRD, SmartLab, Rigaku, Japan). The Brunauer-Emmett-Teller (BET) method was employed to calculate the specific surface area based on the adsorption data in the relative pressure range of 0.05–0.30. The pore size distribution was derived using the Barrett-Joyner-Halenda (BJH) method from the desorption branch of the isotherm. The surface chemical composition and elemental valence states of biochar before and after Cr (VI) adsorption were analyzed using X-ray Photoelectron Spectroscopy (XPS; Thermo Scientific ESCALAB 250Xi, USA) equipped with a monochromatic Al Kα X-ray source (hv = 1486.6 eV). The binding energy scale was calibrated using the C1s peak at 284.8 eV as the reference. Additionally, pH and electrical conductivity (EC) were measured in a 1:10 (w/v) biochar-to-ultrapure water suspension using a portable pH meter (SX-620, Shanghai Sanshin, China), following established protocols. These characterization techniques ensured a comprehensive understanding of the biochar’s structural and chemical properties, critical for its application in mitigating chromium stress in flow-through water systems.

### Flow-through water system design, cultivar selection, and biochar filter application

**Fig. 1:**
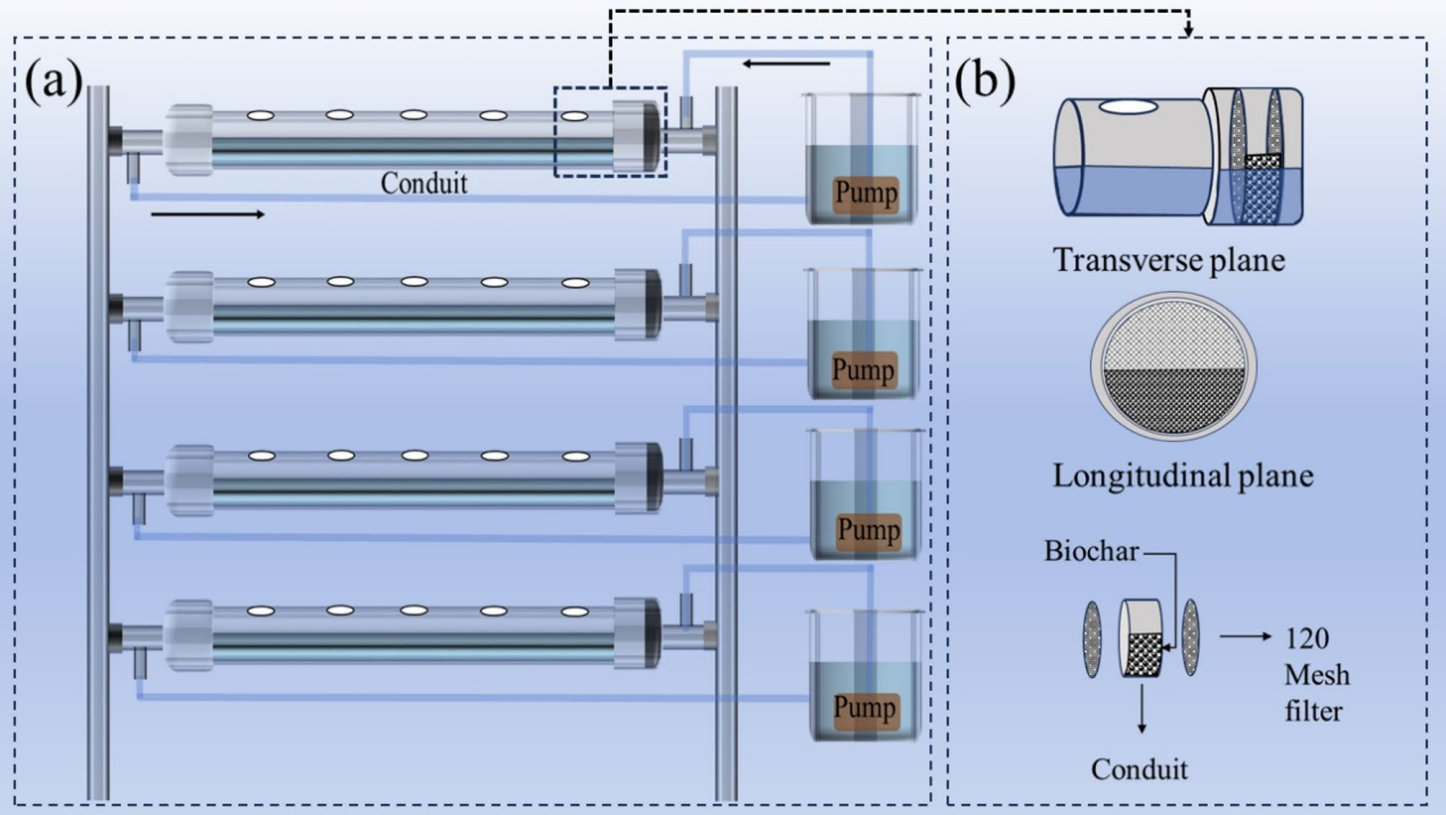
Schematic representation of the biochar filtration system used in the hydroponic setup: (**a**) flow-through hydroponic system with conduit-based plant placement and pump-driven water circulation; (**b**) structural layout of the integrated biochar filtration unit

A custom flow-through hydroponic system was constructed using polyvinyl chloride (PVC) piping to investigate biochar’s efficacy in mitigating chromium stress in Chinese cabbage (*Brassica rapa*) (Fig. 1a-b). The system featured a horizontal channel with evenly spaced perforations (1 cm diameter) to support plant growth, connected to an inlet pipe and a drainage pipe at opposite ends for continuous nutrient solution circulation (Fig. 1a). A submersible pump (16 W, 800 L/h flow rate) ensured consistent delivery of the nutrient solution to the plant roots, maintaining adequate moisture throughout the experiment.

Seeds of the Four-season Cream Bok Choy variety of Chinese cabbage, sourced from Sichuan Province, China, were selected for their disease resistance, stress tolerance, and adaptability [34, 35]. Seeds were sterilized by immersion in 30% (v/v) hydrogen peroxide (H_2_O_2_) for 15 min, followed by triple rinsing with ultrapure water to eliminate residual sterilant. Sterilized seeds were placed on trays lined with moistened filter paper and germinated in an artificial climate chamber (RLD-1000E, Ningbo Ledian, China) under controlled conditions: 10,000 lx light intensity, a 12-hour light/dark cycle with day/night temperatures of 25 °C/20°C, and relative humidity levels of 85%/75%, respectively. After seven days, uniform seedlings were selected and transferred to hydroponic boxes containing nutrient solution. Following a three-day acclimatization period, the seedlings were transplanted into the hydroponic flow-through system for further treatment.

Seedlings were secured in planting baskets using absorbent sponges to protect roots and stabilize their position within the perforated channels. The nutrient solution, circulated from a 2 L storage tank, flowed continuously through the system, delivering nutrients directly to the root zones before returning via the drainage pipe, establishing a closed-loop configuration (Fig. 1a). This design ensured precise control over nutrient and contaminant exposure, consistent with flow-through systems described in prior studies [36].

Biochar filters were installed at the inlet of each hydroponic channel to treat the circulating nutrient solution (Fig. 1b). Each filter consisted of a cylindrical PVC housing fitted with a 120-mesh screen, sized to match the inlet pipe’s internal diameter, ensuring unobstructed flow while retaining biochar particles. Biochar was applied at dosages of 0.5 g/L (BC1), 1.25 g/L (BC2), and 2.5 g/L (BC3), calculated based on the system’s 2 L water capacity per treatment group. Filters were filled with the designated biochar amounts, and each treatment was replicated three times to ensure statistical robustness. A control group without biochar filtration was included for comparison. The hydroponic system operated continuously at ambient temperature (approximately 22–25 °C) for 24 h daily. After a seven-day treatment period under chromium stress (20 mg/L Cr (VI)), plant samples were harvested for growth and physiological analyses.

### Chromium treatment, experimental setup and seed germination assessment

Chromium (Cr) solutions were prepared using potassium dichromate (K_2_Cr_2_O_7_) as the Cr (VI) source to achieve a concentration of 20 mg/L in the flow-through hydroponic system. The required mass of K_2_Cr_2_O_7_ was calculated based on the system’s 2 L water capacity per treatment group, dissolved in deionized water, and stirred continuously until fully solubilized. The resulting Cr solution was stored in a dark, cool environment (4 °C) to prevent photochemical oxidation of Cr (VI) ions, following established protocols [37].

The experimental design comprised five groups: one control (CK) and four treatments (Cr, BC1, BC2, and BC3), each utilizing 2 L of solution. The control group (CK) consisted of deionized water supplemented with Hoagland nutrient solution. The chromium (Cr) treatment group received 20 mg/L of Cr (VI) in combination with Hoagland nutrient solution. The biochar treatment groups (BC1, BC2, and BC3) were exposed to the same Cr (VI) concentration (20 mg/L) and supplemented with biochar at 0.5, 1.25, and 2.5 g/L, respectively, also in Hoagland nutrient solution. Each treatment was replicated thrice to ensure statistical reliability. The experiment was conducted at the laboratory facilities of the School of Ecology, Hainan University, Hainan Province, China (20°3’29"N, 110°19’30"E).

Prior to transplanting into the hydroponic system, a seed germination period and a transitional hydroponic adaptation phase were conducted. Seeds of the Four Seasons Spring Cabbage variety (Chinese cabbage) were germinated in an artificial climate chamber (RLD-1000E, Ledian, Ningbo, China) under controlled conditions: light intensity of 10,000 lx, a day/night temperature cycle of 25 °C/20°C, and relative humidity maintained at 75%. Seven days after germination, uniform seedlings were selected, and their root systems were wrapped with absorbent cotton for protection. The seedlings were then transferred to hydroponic boxes containing nutrient solution for a three-day acclimatization period. Following this, the seedlings were transplanted into the hydroponic flow-through system. Plants were maintained in an environmental laboratory under controlled conditions with temperatures ranging from 26 °C to 30 °C and relative humidity maintained at approximately 70%, allowing for stable growth prior to chromium exposure and biochar treatment.

### Index measurements

#### Assessment of growth parameters

Growth parameters of Chinese cabbage (*Brassica rapa*) seedlings were assessed after seven days of cultivation under five treatment conditions. Seedlings were harvested and separated into shoots and roots using sterilized scissors, followed by thorough rinsing with ultrapure water to remove residual dust and metal ions. Fresh biomass of shoots and roots was immediately weighed using a precision balance and recorded for each replicate.

#### Assessment of key physiological indices

Physiological responses of Chinese cabbage to chromium stress and biochar filtration were evaluated through multiple enzymatic and biochemical assays. Peroxidase (POD) activity was measured following the guaiacol oxidation method of [38], with absorbance recorded at 470 nm. Superoxide dismutase (SOD) activity was determined using the nitroblue tetrazolium (NBT) reduction protocol by [39], monitored at 560 nm. Catalase (CAT) activity was quantified based on H_2_O_2_ decomposition, as described by [40], with absorbance measured at 240 nm. Hydrogen peroxide (H_2_O_2_) content in plant tissues was assessed using the titanium sulfate method of [41], with absorbance at 415 nm.

Protein content was determined using the Coomassie Brilliant Blue G-250 dye-binding method of [42], with 0.1 g of fresh plant tissue homogenized and absorbance measured spectrophotometrically at 650 nm. Soluble sugar content was determined using the anthrone colorimetric method described by [43], with 0.5 g of fresh tissue and absorbance measured at 420 nm. Proline content was quantified following the ninhydrin-based assay outlined by [44], using 0.1 g of fresh tissue and measuring absorbance at 490 nm. Malondialdehyde (MDA) levels, indicative of lipid peroxidation, were assessed using the thiobarbituric acid (TBA) method described by [45], with absorbance recorded at 532 nm and corrected for nonspecific turbidity at 600 nm.

Photosynthetic pigments were extracted using 80% acetone. Extracts were centrifuged at 4000×g for 5 min at 4 °C, and chlorophyll concentrations (chlorophyll a and b) were determined according to [46], with absorbance measured at 663 nm and 645 nm. Carotenoid content was quantified from the same acetone extracts, with absorbance recorded at 470 nm following [47]. Elemental composition of plant tissues was analyzed using inductively coupled plasma-optical emission spectrometry (ICP-OES), as adapted from [48], after acid digestion of dried samples [26].

### Determination of Cr concentration

Chromium (Cr) content in plant tissues was quantified using graphite furnace atomic absorption spectrometry (GFAAS) following GB/T 5009.123–2023 standard protocol. Harvested plant samples were oven-dried at 60 °C, ground into a fine powder, and subjected to acid digestion (HNO_3_:HClO_4_, 4:1 v/v). Digests were analyzed for Cr concentration at a wavelength of 357.9 nm using a GFAAS instrument (Hitachi Z-2000 Series, Japan). Calibration curves were prepared from standard Cr solutions (0–100 µg/L), ensuring accurate quantification across the concentration range. Each measurement was performed in triplicate to enhance precision.

### Correlation matrix analysis

To explore interrelationships among measured physiological, biochemical, and growth-related traits, a Pearson correlation matrix was computed. Correlation coefficients were visualized using a heatmap generated in Python with the Seaborn and Matplotlib libraries. Positive and negative correlations were color-coded (e.g., red for positive, blue for negative), with clustering patterns used to interpret trait co-responsiveness to chromium stress and biochar treatment.

### Hierarchical clustering analysis (HCA)

To classify treatment responses based on the similarity of physiological and biochemical traits, hierarchical clustering analysis (HCA) was performed. The clustering was applied to trait means for each treatment group. A dendrogram was generated using Python’s SciPy and Matplotlib libraries to illustrate the relative distance and similarity among treatment profiles.

### Radar chart analysis

To compare the physiological, biochemical, and stress-related responses of Chinese cabbage across treatments, radar (spider) charts were constructed using normalized mean values of selected shoot and root traits. Trait data were grouped by treatment and averaged. For shoot traits, parameters included biomass (WeightS), length (LengthS), photosynthetic pigments (ChllaS, ChllbS, TchllS, CarotenoidsS), oxidative stress markers (MDAS, H_2_O_2_S), and antioxidant enzyme activities (SODS, PODS, CATS). For root traits, the included parameters were root biomass (RWeight), length (RootLength), chromium accumulation (RCr), lipid peroxidation (RMDA), hydrogen peroxide (RH_2_O_2_), and antioxidant enzymes (RSOD, RPOD, RCAT, RPRO). Radar plots were generated using Python (Matplotlib library), with each treatment represented as a polygon, enabling visual comparison of trait profiles and treatment effects.

### Statistical analysis

Experimental data were compiled and processed using Microsoft Excel 2016. Statistical analyses were conducted with IBM SPSS Statistics Version 26 (IBM Corporation, USA). One-way analysis of variance (ANOVA) was employed to evaluate differences among treatment groups (CK, Cr, BC1, BC2, BC3), followed by Tukey’s Honest Significant Difference (HSD) post-hoc test to identify specific pairwise differences. Statistical significance was determined at a threshold of *p* < 0.05, consistent with standard practices in biochar remediation studies [26]. Radar graphs were generated using Python’s Matplotlib library. Additionally, Origin Lab 2025 was used for figure generation, while statistical data visualizations and plant tissue images were compiled and arranged using Adobe Photoshop 2024 and Microsoft PowerPoint 2016.

## Results and discussion

### Characterization of biochar

**Fig. 2:**
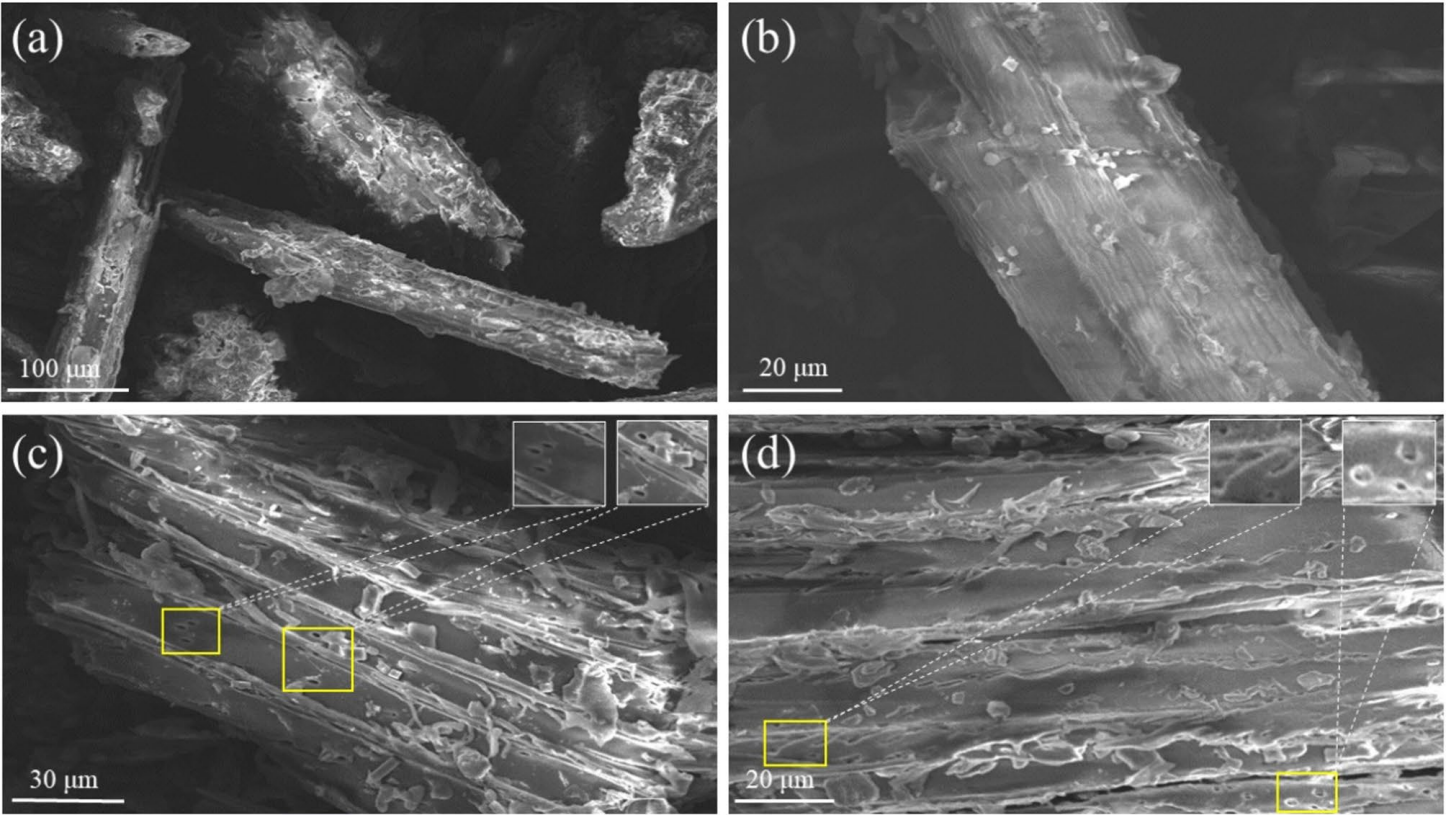
Scanning Electron Microscopy (SEM) images of biochar before and after chromium adsorption. (**a**) Low-magnification SEM image showing the rough, porous surface morphology of pristine biochar. (**b**) Higher magnification image revealing fibrous structures and abundant adsorption sites. (**c**) SEM image of biochar post-Cr adsorption, with visible chromium deposits localized in porous regions (highlighted in yellow boxes). (**d**) SEM evidence of chromium accumulation on the biochar surface, with inset images showing Cr deposition within micropores

The surface morphology of biochar derived from *Ageratina adenophora* was characterized using scanning electron microscopy (SEM), revealing a rough, heterogeneous texture (Fig. 2a, b). Higher magnification images (Fig. 2c, d) disclosed a network of micropores and mesopores, significantly enhancing the specific surface area available for adsorption. These structural features are critical for the effective capture of Cr ions, reducing their bioavailability in aqueous systems and mitigating toxicity to plants [49]. Pre-adsorption SEM images (Figure S1b) reveal a folded, porous surface with loosely structured cavities, whereas post-adsorption images (Figure S2b) confirm the preservation of micropores and mesopores within these surface folds. The preservation of this porous architecture during Cr adsorption underscores the physical stability of biochar, a key factor in its sustained adsorption capacity [50].

EDS mapping (Figure S2e) and spectral analysis (Figure S2a) further confirmed chromium adsorption, with Cr signals localized within the biochar’s folds, micropores, and cavities. Elemental composition data (Table S1) show that the pre-adsorption biochar was primarily composed of 68.2% carbon (C) and 31.6% oxygen (O). Post-adsorption, the composition shifted to 66.3% C, 33.4% O, and 0.2% Cr, reflecting successful Cr uptake. The slight decrease in carbon content may be attributed to Cr occupying active sites or interacting with carbon-containing functional groups, potentially leading to their chemical transformation or detachment [51, 52]. Prolonged exposure to aqueous conditions during the adsorption process may also induce minor structural alterations in the biochar, potentially contributing to carbon loss [53]. Conversely, the increase in oxygen content likely results from the formation of oxygen-rich functional groups (e.g., -OH, -COOH) during Cr adsorption, which may complex with Cr ions [54]. A potential redox reaction between biochar’s oxygen-containing groups and Cr (VI) could further elevate oxygen levels by reducing Cr (VI) to Cr (III), a process consistent with prior studies [55, 56].

**Fig. 3:**
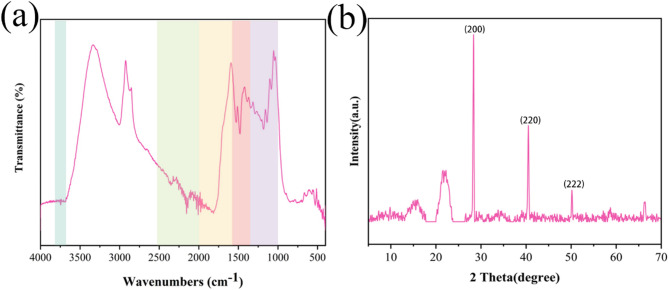
(**a**) FTIR and (**b**) XRD patterns of biochar

FTIR analysis (Fig. 3a) elucidated the functional groups on biochar’s surface, critical to its Cr adsorption capacity. A peak at 3732.74 cm^− 1^ (3850–3660 cm^− 1^ range) corresponds to -OH stretching vibrations, indicative of residual moisture or hydroxyl groups post-pyrolysis [57]. The peak at 2999.49 cm^− 1^ (2900–3000 cm^− 1^) reflects C-H stretching in aromatic or aliphatic structures, affirming the organic nature of the biochar [58]. Peaks between 2364.46 and 2152.03 cm^− 1^ (2500–2000 cm^− 1^) suggest C ≡ C or C ≡ N stretching, or C-H bending, aligning with findings by [59]. Carbonyl (C = O) stretching vibrations at 1957.86 and 1818.52 cm^− 1^ (1960–1650 cm^− 1^) indicate groups capable of coordinating with Cr ions, enhancing adsorption [60]. Aromatic C = C stretching or C-H bending at 1526.14 and 1475.88 cm^− 1^ (1675–1500 cm^− 1^) points to structures that bolster surface area and adsorption potential [61]. Strong C-O stretching peaks at 1176.64, 1130.96, and 1076.14 cm^− 1^ (1300–1000 cm^− 1^) are attributed to ether and alcohol groups, key contributors to Cr binding [27].

XRD analysis (Fig. 3b) revealed the crystalline structure of the biochar, with a prominent diffraction peak at 28.345° (d = 3.1460 Å), corresponding to the (200) plane, indicating a high degree of crystallinity [62]. Peaks at 40.5° (d = 2.2251 Å; (220) plane) and 50.18° ((222) plane, quartz-related) suggest a cubic crystalline lattice (a ≈ 6.2917 Å), consistent with plant-derived biochar [61]. These crystalline phases, combined with the functional groups identified via FTIR, underscore biochar’s multifaceted nature, enhancing its mineral composition and interaction with Cr ions in hydroponic systems. The synergy of hydroxyl, carbonyl, and crystalline structures positions biochar as an effective medium for heavy metal remediation [26].

The Brunauer-Emmett-Teller (BET) surface area and porosity characteristics of the biochar (BC), determined using nitrogen adsorption-desorption isotherms at 77 K, are presented in Table S2 and Figure S3. These results highlight the structural properties of BC relevant to its adsorption performance. Further details are provided in Supplementary Sect. 1.

In addition, the C1s XPS spectra of biochar before and after Cr (VI) adsorption are shown in Figure S4 and summarized in Table S3. These results provide insights into changes in surface carbon bonding environments associated with chromium removal. Detailed interpretations can be found in Supplementary Sect. 2.

### Effect of biochar filters on the physiological traits of Chinese cabbage exposed to chromium stress

**Fig. 4:**
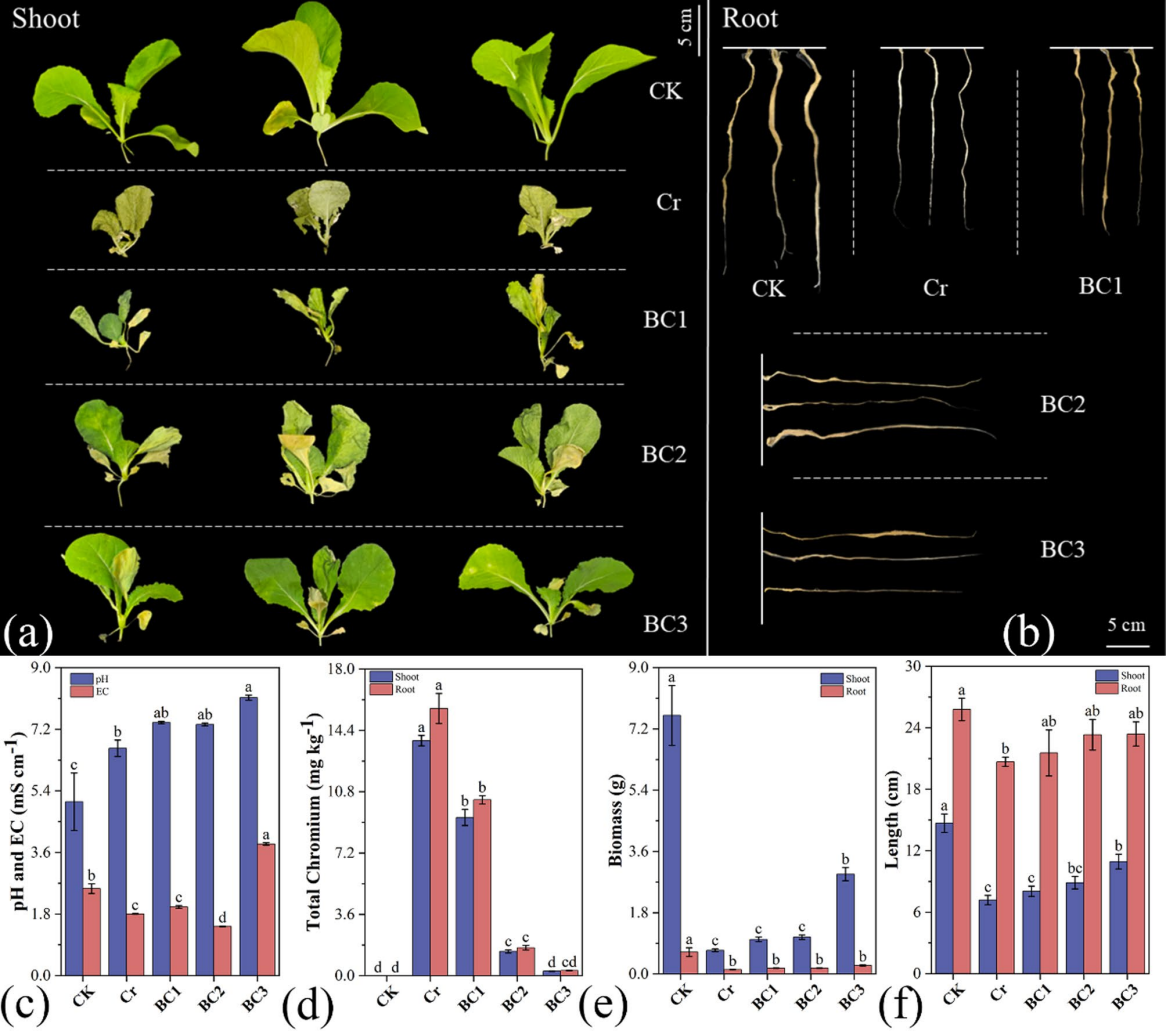
Growth performance and physiological responses of Chinese cabbage under different treatments. (**a**) Shoot morphology, (**b**) Root morphology, (**c**) pH and electrical conductivity (EC), (**d**) Total chromium accumulation in shoot and root tissues, (**e**) Shoot and root biomass, (**f**) Shoot and root length. Treatments: CK (control), Cr (chromium stress), BC1 (0.5 g/L biochar), BC2 (1.25 g/L biochar), and BC3 (2.5 g/L biochar). Data are presented as mean ± standard deviation (*n* = 3). Error bars represent standard deviation. Different lowercase letters indicate statistically significant differences at *P* < 0.05

Chromium (Cr) exposure had a negative impact on Chinese cabbage (*Brassica rapa*), as demonstrated by visible stress symptoms and a significant reduction in biomass (Fig. 4a-b). Biochar filtration, however, mitigated these impacts, enhancing plant resilience across multiple physiological parameters.

#### Effects on pH and electrical conductivity (EC)

The influence of Cr and biochar treatments on water pH and EC during Chinese cabbage cultivation is illustrated in Fig. 4c. Relative to the control (CK), the Cr treatment increased pH by 30.77% and decreased EC by 29.07%. The elevated pH likely results from the formation of chromate ions (CrO_4_^2−^) under Cr (VI) exposure, which increases solution alkalinity [63], coupled with cation exchange reducing H^+^ ions [64]. The decline in EC may reflect Cr interference with nutrient uptake, lowering concentrations of ions such as Ca^2+^, Mg^2+^, and K^+^ in the solution [65].

Biochar treatments showed dose-dependent effects. In comparison to the control (CK), the BC1 (0.5 g/L) and BC2 (1.25 g/L) treatments increased pH by 45.67% and 44.55%, respectively, while reducing electrical conductivity (EC) by 21.05% and 43.46%. In contrast, the BC3 (2.5 g/L) treatment markedly elevated pH by 59.90% and increased EC by 51.34%. The pH rise across biochar treatments aligns with biochar’s inherent alkalinity [66], while EC reductions in BC1 and BC2 likely stem from biochar’s adsorption of ions, altering their availability [67]. The elevated EC in BC3 may indicate saturation of adsorption sites or release of biochar-derived ions (e.g., Na^+^, K^+^), influencing solution conductivity.

#### Effects on Cr content and plant biomass

Chromium accumulation was highest in the Cr treatment group, with significantly greater retention in roots than in shoots (Fig. 4d), indicating that the roots function as a primary barrier to Cr translocation within the plant [68]. Biochar significantly reduced Cr uptake. In the BC1 treatment, Cr content decreased by 32.68% in shoots and 34.22% in roots. The BC2. and BC3 treatments exhibited more substantial reductions: 89.64% and 98.03% in shoots, and 89.55% and 98.04% in roots, respectively. This reduction is attributed to biochar’s porous structure and functional groups (e.g., -OH, -COOH), which bind Cr ions via electrostatic interactions and complexation, reducing bioavailability [69]. XPS and FTIR analyses corroborate these mechanisms, highlighting biochar’s capacity to form stable Cr complexes.

Biochar treatments also enhanced plant biomass (Fig. 4e). Compared to the Cr group, shoot biomass increased by 46.40%, 55.76%, and 322.48% in BC1, BC2, and BC3, respectively, while root biomass rose by 32.56%, 34.11%, and 93.80%. Shoot and root lengths similarly improved (Fig. 4f), with increases of 11.98%, 23.68%, and 52.37% in shoots, and 4.19%, 12.76%, and 13.14% in roots across BC1, BC2, and BC3. Chromium, particularly Cr (VI), disrupts cellular division, elongation, and nutrient uptake, leading to biomass declines [70]. The mitigation of chromium-induced stress by biochar is likely attributed to reduced Cr toxicity and improved nutrient availability, as reflected by the enhanced growth parameters [71]. The dose-dependent response, most pronounced in BC3, underscores biochar’s potential as a remediation tool in hydroponic systems.

#### Effects on photosynthetic pigments

**Fig. 5:**
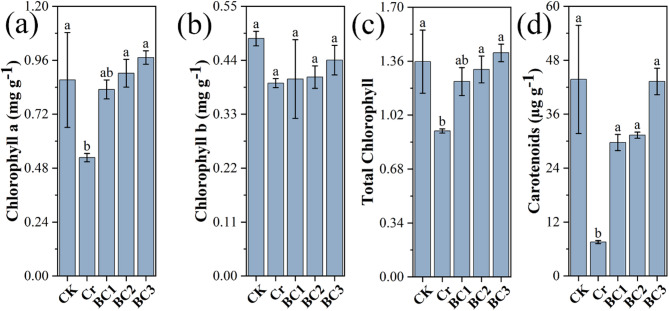
Effects of chromium stress and biochar treatment on photosynthetic pigments in Chinese cabbage. (**a**) Chlorophyll a content, (**b**) Chlorophyll b content, (**c**) Total chlorophyll content, and (**d**) Carotenoid content across different treatments

Photosynthetic pigments, including chlorophyll a, chlorophyll b, total chlorophyll, and carotenoids, are critical for plant growth, reproduction, and stress adaptation [72]. Carotenoids play a crucial role in protecting plants from photooxidative damage by scavenging reactive oxygen species (ROS) and stabilizing lipophilic cellular structures [73]. Chromium (Cr) stress significantly impaired these pigments in Chinese cabbage (*Brassica rapa*), as shown in Fig. 5. Compared to the control (CK), the Cr treatment reduced chlorophyll a, chlorophyll b, total chlorophyll, and carotenoid content, reflecting inhibited photosynthesis. This decline is attributed to Cr-induced ROS overproduction, which disrupts chlorophyll biosynthesis, degrades photosynthetic enzymes, and damages chloroplast ultrastructure [74–76].

Biochar application alleviated the adverse effects of chromium stress in a dose-dependent manner. In the BC1 treatment (0.5 g/L biochar), levels of chlorophyll a, chlorophyll b, total chlorophyll, and carotenoids increased by 57.58%, 2.11%, 33.86%, and 294.45%, respectively, compared to the Cr-stressed group. The BC2 treatment (1.25 g/L) further enhanced these pigments by 71.26%, 3.11%, 42.13%, and 316.14%, while the BC3 treatment (2.5 g/L) yielded the greatest improvements: 84.46%, 4.67%, 53.52%, and 357.35%, respectively. These gains suggest biochar reduces Cr bioavailability through adsorption, mitigating oxidative stress and supporting pigment synthesis [77]. The pronounced effect in BC3 likely reflects enhanced root health, nutrient uptake, and antioxidant capacity, minimizing ROS damage and boosting photosynthetic efficiency [78]. These findings underscore biochar’s potential to remediate Cr-stressed hydroponic systems.

#### Effects on soluble protein and sugar

Soluble proteins and sugars serve as osmoregulatory compounds, maintaining cellular homeostasis and mitigating oxidative damage under stress [79, 80]. Chromium stress significantly reduced soluble protein content in Chinese cabbage shoots by 53.61% compared to CK (Fig. S5a), reflecting Cr toxicity. This decline likely results from ROS-induced protein damage, disrupted synthesis, and activation of proteolytic pathways (e.g., lysosomal proteases, proteasomes), alongside impaired folding and repair mechanisms [81–83]. Biochar treatments reversed this effect, increasing shoot protein content by 50.35%, 72.39%, and 147.80% in BC1, BC2, and BC3, respectively, relative to the Cr group. The BC3 treatment’s superior efficacy suggests biochar’s adsorption of Cr ions alleviates toxicity, supporting protein metabolism [84, 85].

Similarly, chromium stress led to a reduction in soluble sugar content by 58.92% in shoots and 35.45% in roots compared to the control (CK) (Figure S5b), likely due to impaired photosynthesis and inhibited RuBisCO activity, which restricts CO_2_ fixation [86]. Reduced stomatal conductance and disrupted carbon metabolism further diminish sugar production [81, 87]. Biochar treatments effectively counteracted these effects, increasing shoot soluble sugar content by 28.97%, 39.71%, and 62.66% in BC1, BC2, and BC3, respectively, and root sugar content by 3.38%, 36.60%, and 42.94%. The substantial improvement observed in the BC3 treatment corresponds with enhanced photosynthetic capacity and reduced chromium uptake, thereby promoting sugar synthesis and accumulation [88]. These results highlight biochar’s role in restoring metabolic balance under Cr stress, with higher doses optimizing plant resilience.

#### Effects on nutrient contents

Chromium (Cr) exposure significantly impaired nutrient uptake in Chinese cabbage (*Brassica rapa*), as evidenced by reduced levels of essential elements in the Cr treatment group compared to the control (CK) (Fig. S6). Nitrogen (N) decreased by 5.69%, phosphorus (P) by 40.37%, potassium (K) by 8.53%, calcium (Ca) by 25.14%, and magnesium (Mg) by 28.97%. These reductions reflect chromium-induced damage to root structures and the competitive inhibition of nutrient uptake, ultimately compromising overall plant growth [64, 82, 89].

Biochar application mitigated these effects, with nutrient recovery increasing in a dose-dependent manner. In the BC1 treatment (0.5 g/L biochar), N, P, K, Ca, and Mg contents rose by 4.58%, 5.20%, 7.47%, 21.56%, and 0.40%, respectively, relative to the Cr group. The BC2 treatment (1.25 g/L) further enhanced levels by 8.91% (N), 20.99% (P), 10.02% (K), 20.35% (Ca), and 10.63% (Mg), while BC3 (2.5 g/L) yielded the greatest improvements: 20.82% (N), 37.11% (P), 24.95% (K), 6.14% (Ca), and 22.33% (Mg). This progressive recovery is consistent with biochar’s ability to adsorb chromium, thereby reducing its bioavailability and improving root function and nutrient uptake [90, 91]. The superior performance of the BC3 treatment suggests the presence of an optimal threshold for biochar-mediated remediation under Cr stress [92].

Nutrient availability underpins key physiological processes: N supports protein and chlorophyll synthesis, P drives energy metabolism and root growth, K regulates stomatal and enzymatic functions, Ca stabilizes cell walls, and Mg anchors photosynthesis [93, 94]. Cr disrupts these processes via ROS-induced stress and ionic competition [95], but biochar’s ameliorative effects enhance rhizosphere conditions, facilitating nutrient uptake and plant resilience.

#### Effects on antioxidant enzymes

**Fig. 6:**
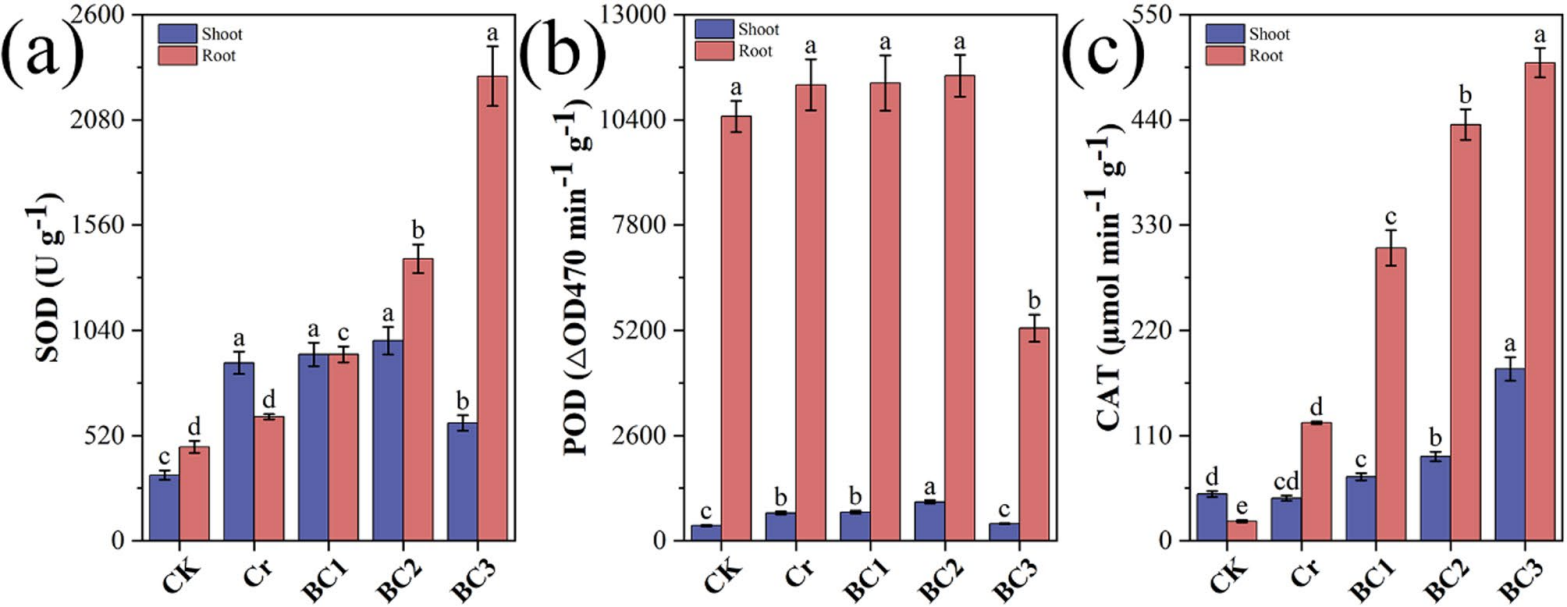
Effects of chromium stress and biochar treatment on antioxidant enzyme activities in the shoots and roots of Chinese cabbage. (**a**) Superoxide dismutase (SOD) activity, (**b**) Peroxidase (POD) activity, (**c**) catalase (CAT) activity across different treatments

Antioxidant enzyme activities in Chinese cabbage, including superoxide dismutase (SOD), peroxidase (POD), and catalase (CAT), were significantly altered under Cr stress and biochar treatments (Fig. 6c). Compared to CK, the Cr group exhibited a 171.30% increase in SOD, an 83.44% rise in POD, and an 8.98% decrease in CAT. Cr-induced ROS overproduction triggers oxidative damage, prompting elevated SOD and POD activities to neutralize superoxide and H_2_O_2_, respectively [96, 97]. The decline in CAT activity may indicate enzymatic suppression in response to severe stress conditions [98, 99].

Biochar treatments modulated these responses variably. In shoots, BC1 increased SOD, POD, and CAT by 4.73%, 83.65%, and 19.38%, respectively, relative to the Cr group, while BC2 enhanced them by 7.93%, 93.27%, and 35.96%. Conversely, BC3 reduced SOD by 33.88% but boosted POD by 13.57% and CAT by 303.79%. In roots, Cr elevated SOD, POD, and CAT by 32.26%, 7.44%, and 506.94%, respectively, compared to CK. Biochar further increased these in BC1 (50.35%, 7.84%, 147.80%) and BC2 (127.13%, 9.60%, 252.42%), but BC3 showed a 274.34% rise in SOD, a 304.80% increase in CAT, and a 49.87% drop in POD. These patterns suggest biochar enhances antioxidant defenses at lower doses (BC1, BC2), while high doses (BC3) induce complex shifts [85, 100]. The reduction in SOD activity observed in BC3 shoot tissues may be attributed to biochar binding metal cofactors such as Cu and Zn, which are essential for SOD functionality [101], or to conformational alterations that impair enzyme activity [102]. Alternatively, biochar may alter hormonal signaling, prioritizing POD and CAT to scavenge H_2_O_2_ [103, 104]. In roots, the BC3 POD decline could reflect similar cofactor competition or a threshold effect at high biochar levels [105, 106]. These findings align with concentration-dependent enzyme responses reported by [104], highlighting biochar’s dual role in alleviating Cr stress while potentially inhibiting specific enzymes at higher doses [107].

#### Effects on H_2_O_2_, MDA, and proline

**Fig. 7:**
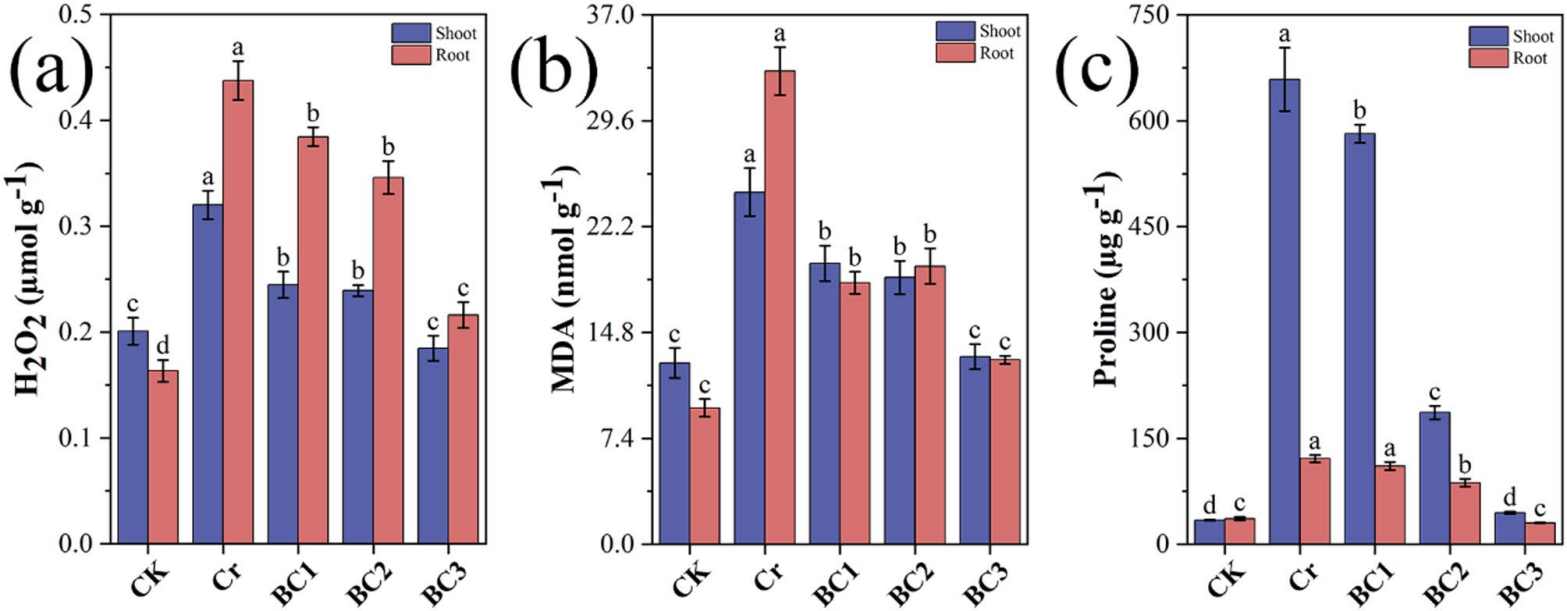
Effects of chromium stress and biochar treatment on (**a**) Hydrogen peroxide (H_2_O_2_) content, (**b**) Malondialdehyde (MDA) content, and (**c**) Proline content in shoot and root tissues under different treatments

Chromium (Cr) stress significantly altered hydrogen peroxide (H_2_O_2_), malondialdehyde (MDA), and proline (PRO) levels in Chinese cabbage (*Brassica rapa*), reflecting oxidative and physiological responses (Fig. 7a). Compared to the control (CK), the Cr treatment increased shoot H_2_O_2_, MDA, and PRO by 59.37%, 94.30%, and 1828.62%, respectively, with roots showing greater sensitivity at 167.62%, 246.80%, and 246.80%. These elevated levels reflect chromium-induced reactive oxygen species (ROS) generation, lipid peroxidation, and osmotic adjustment, with effects particularly pronounced in roots due to their direct exposure to Cr stress [108].

Biochar application markedly reduced these stress markers, with effects intensifying at higher doses. In the BC3 treatment (2.5 g/L biochar), shoot H_2_O_2_, MDA, and PRO decreased by 42.31%, 46.69%, and 93.20%, respectively, relative to the Cr group, while roots showed reductions of 50.59%, 41.24%, and 61.03%. Lower H_2_O_2_ levels suggest biochar enhances antioxidant defenses, curbing ROS accumulation [61]. Reduced MDA reflects diminished lipid peroxidation and improved membrane integrity, while the substantial PRO decline in BC3 indicates alleviated stress, as PRO accumulates as an Osmo protectant and antioxidant under Cr toxicity [109]. These findings are consistent with biochar’s ability to adsorb chromium and promote root health, thereby enhancing overall plant tolerance to Cr stress [84, 110].

### Statistical correlation of physiological, biochemical, and morphological parameters

**Fig. 8:**
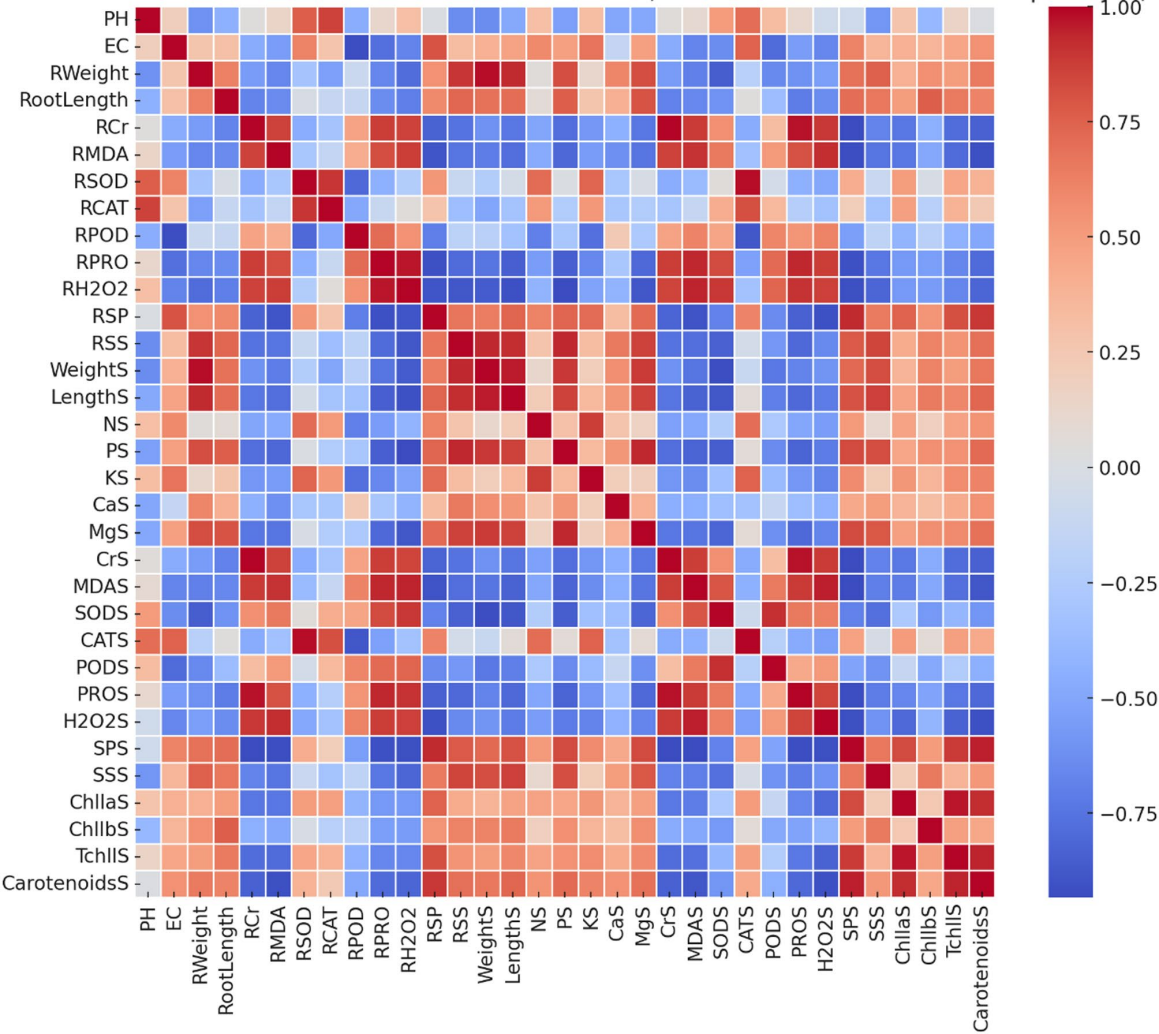
Correlation matrix illustrating relationships between physiological, biochemical, and growth parameters of Chinese cabbage under chromium stress and biochar treatment. Red indicates positive correlations; blue represent negative correlations

The correlation matrix (Fig. 8) elucidates relationships among physiological, biochemical, and growth parameters under Cr stress and biochar treatment. Chromium accumulation in roots (RCr) exhibited a strong negative correlation with growth metrics (root weight, shoot weight, root length), confirming Cr’s inhibitory effect on development [111]. Oxidative stress markers (H_2_O_2_, MDA) positively correlated with RCr, underscoring Cr-induced oxidative damage, while their association with antioxidant enzymes (SOD, POD, CAT) reflects activated defense mechanisms. Variability in enzyme correlations suggests differential responses influenced by Cr levels and biochar dosage [112].

Photosynthetic pigments (chlorophyll a, b, carotenoids) showed strong positive correlations with biomass and shoot length, linking photosynthetic capacity to plant vigor, and negative correlations with H_2_O_2_ and MDA, indicating ROS-mediated pigment degradation under Cr stress. Nutrient elements (N, P, K, Ca, Mg) positively correlated with growth parameters, highlighting their role in counteracting Cr toxicity. Biochar-treated samples displayed enhanced nutrient uptake, likely due to improved hydroponic conditions and reduced Cr bioavailability, supporting plant resilience.

### Hierarchical clustering analysis (HCA)

**Fig. 9:**
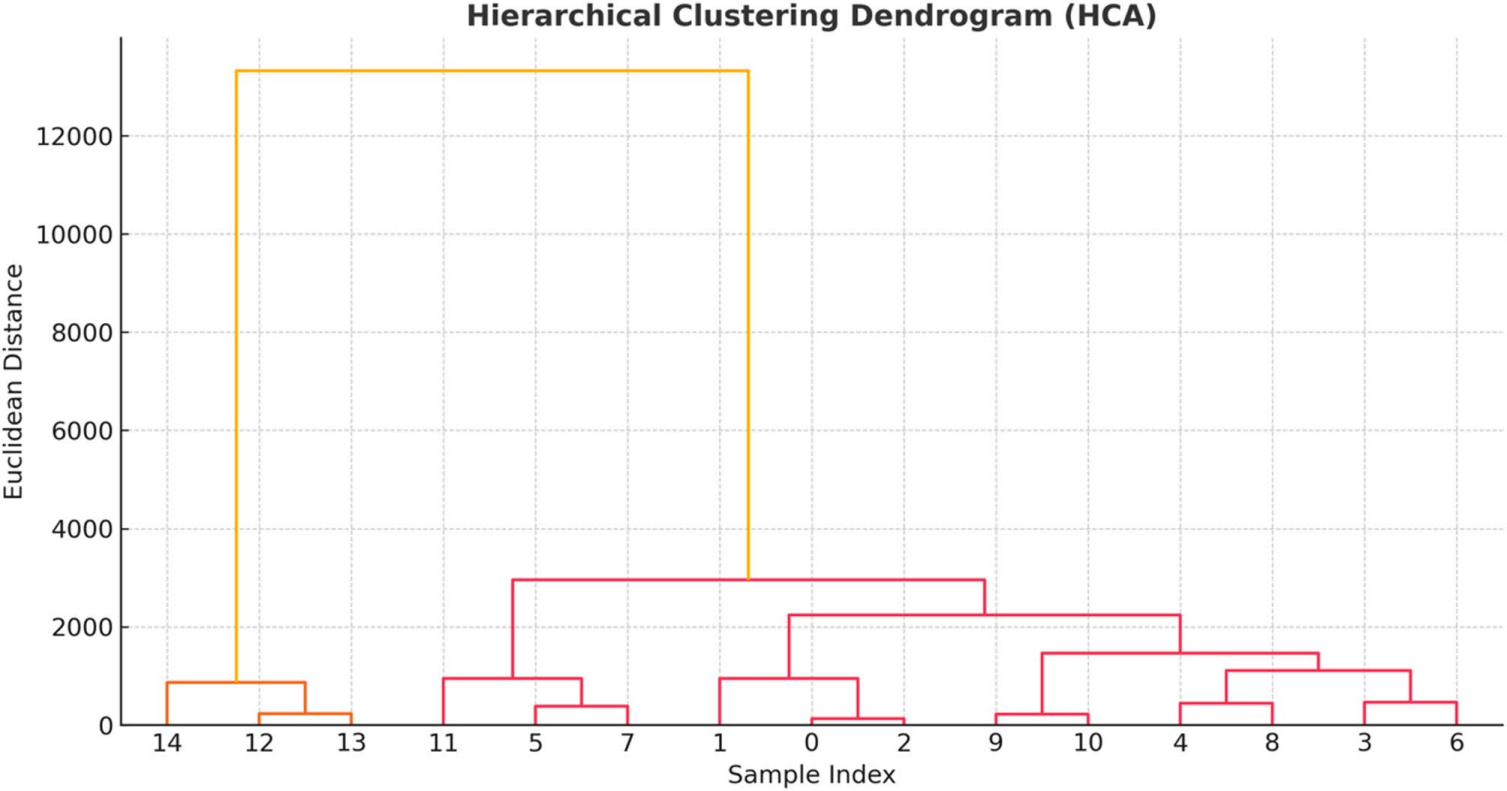
Hierarchical clustering dendrogram (HCA) of physiological, biochemical, and growth parameters in Chinese cabbage under chromium stress and biochar filtration

Hierarchical Clustering Analysis (HCA) (Fig. 9) revealed clear separation among treatment groups based on their physiological, biochemical, and growth response profiles. Cr-stressed samples without biochar formed separate clusters, characterized by elevated H_2_O_2_, MDA, and reduced nutrient uptake, reflecting severe toxicity [113]. In contrast, biochar-treated samples (BC1, BC2, BC3) clustered together, exhibiting consistent mitigation effects: enhanced antioxidant activity, nutrient accumulation, and biomass. This clustering pattern underscores biochar’s dose-dependent amelioration of Cr stress, aligning with its structural and functional properties and reinforcing its potential as a remediation tool in hydroponic systems [114].

### Radar chart analysis of morphological and physiological traits

**Fig. 10:**
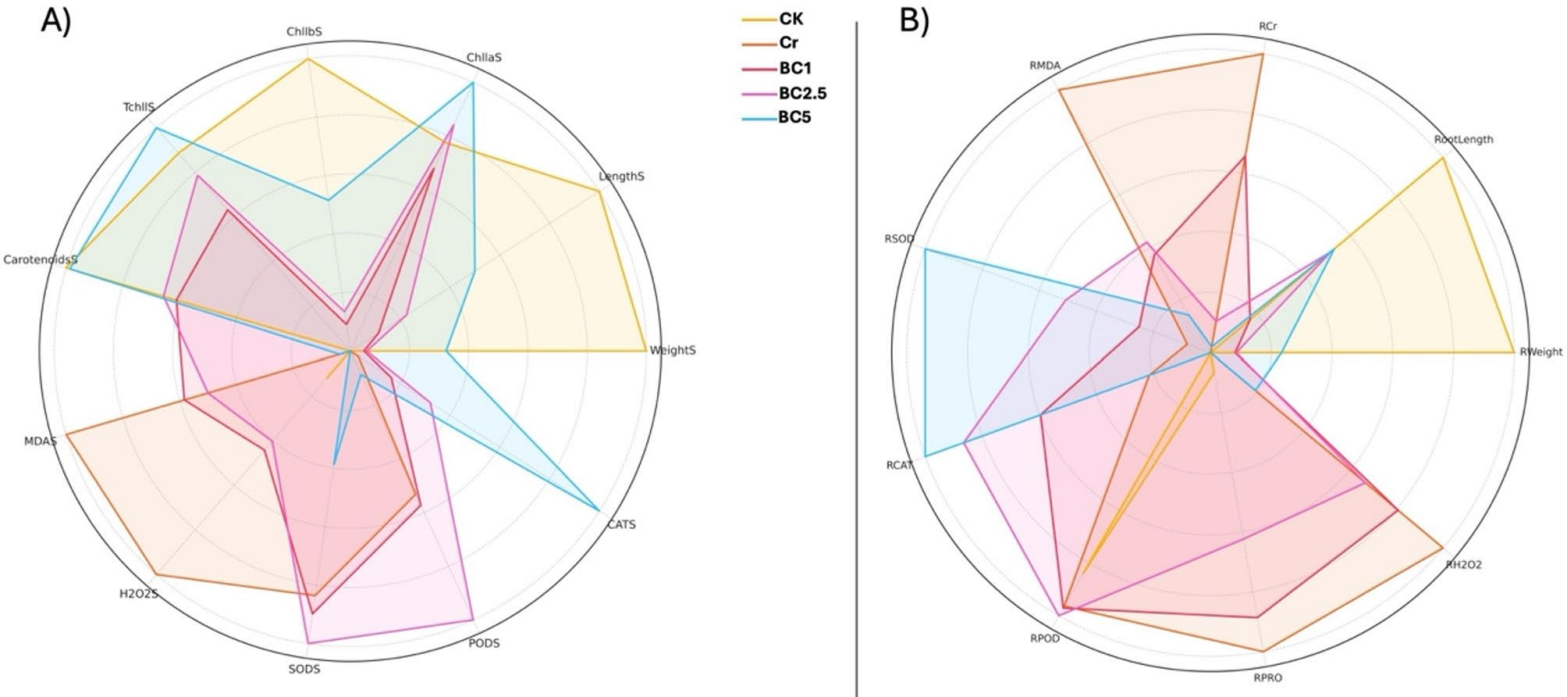
Radar chart comparison of normalized physiological and biochemical trait profiles of Chinese cabbage under chromium stress and biochar treatment. (**a**) Shoot traits include biomass (WeightS), length (LengthS), chlorophyll pigments (ChllaS, ChllbS, TchllS), carotenoids (CarotenoidsS), oxidative stress markers (MDAS, H2O2S), and antioxidant enzyme activities (SODS, PODS, CATS). (**b**) Root traits include biomass (RWeight), length (RootLength), chromium accumulation (RCr), oxidative stress indicators (RMDA, RH2O2), and antioxidant enzyme activities (RSOD, RCAT, RPOD, RPRO)

Radar chart visualizations (Figs. 10a&b) provide a comparative overview of physiological, biochemical, and stress-related traits in Chinese cabbage under chromium stress and biochar treatment [115]. Normalization of trait values revealed distinct patterns demonstrating the effectiveness of biochar in mitigating chromium toxicity at both shoot and root levels.

In the shoot radar chart (Fig. 10a), chromium stress (Cr) led to marked declines in biomass (WeightS), chlorophyll pigments (ChllaS, ChllbS, TchllS), and carotenoids, while increasing oxidative stress markers such as MDA and H₂O₂. These trends underscore the phytotoxic effects of chromium, which impair photosynthesis and elevate reactive oxygen species (ROS) accumulation. Conversely, biochar treatments (especially BC3) demonstrated improved performance across nearly all traits. Notably, BC3-treated plants showed enhanced chlorophyll content and elevated antioxidant enzyme activities (SOD, POD, CAT), indicating effective alleviation of chromium-induced oxidative stress. The radar plot visually confirms that biochar application supports shoot-level physiological stability under metal stress.

The root radar chart (Fig. 10b) further reveals the severity of chromium toxicity and biochar’s ameliorative effects belowground. Chromium treatment caused substantial increases in MDA, H₂O₂, and chromium accumulation (RCr), while simultaneously reducing biomass (RWeight), root length, and antioxidant activity. In contrast, BC3-treated roots displayed reduced Cr uptake and oxidative markers, along with improved root biomass and elevated antioxidant responses (RSOD, RCAT, RPOD, RPRO). These shifts reflect biochar’s ability to immobilize heavy metals, improve nutrient availability, and enhance the antioxidant defense system at the root level.

Collectively, the radar charts illustrate that biochar not only mitigates chromium-induced stress but also enhances physiological resilience by promoting pigment synthesis, reducing oxidative damage, and improving root–shoot coordination. The observed dose-dependent responses-with BC3 outperforming lower concentrations-underscore the importance of optimizing biochar application rates to achieve maximum remediation efficacy.

### Proposed mechanism of biochar-mediated chromium detoxification in Chinese cabbage

**Fig. 11:**
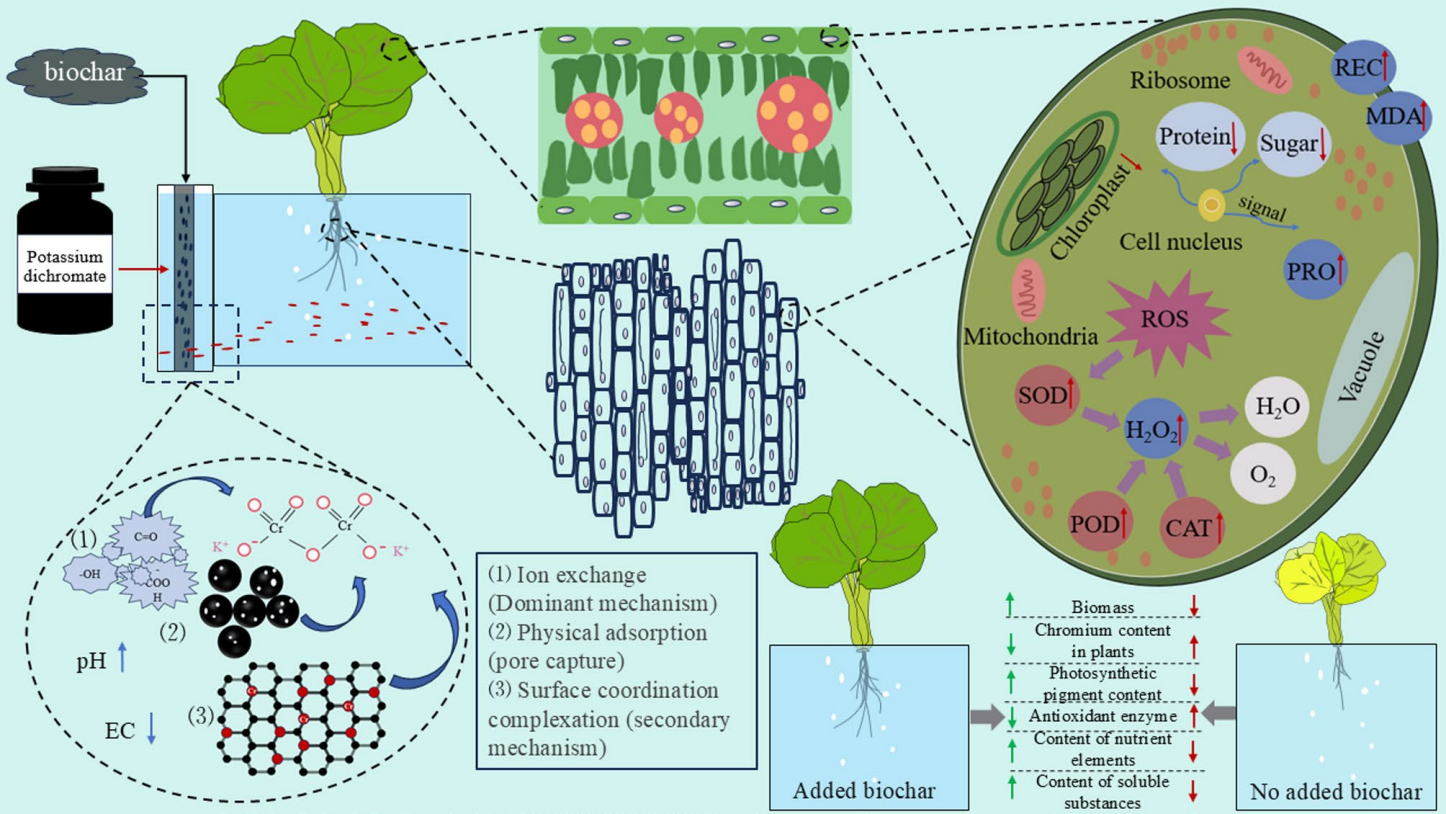
Proposed mechanism of biochar-mediated mitigation of chromium (Cr) toxicity in Chinese cabbage (*Brassica rapa*) grown in a flow-through water system

Figure 11 illustrates the proposed mechanism by which biochar mitigates chromium (Cr) toxicity in *Brassica rapa* cultivated in a flow-through water system. Acting as a filtration medium, the biochar immobilizes Cr primarily through ion exchange, physical adsorption within its porous matrix, and surface complexation with functional groups. BET analysis confirmed the biochar’s mesoporous structure and high specific surface area, which facilitate effective Cr (VI) entrapment via pore filling and diffusion mechanisms. XPS analysis further revealed the coexistence of Cr (VI) and Cr (III) species on the biochar surface after treatment, indicating partial reduction of Cr (VI) to the less toxic Cr (III), likely mediated by redox-active oxygen-containing groups. This redox activity, in conjunction with strong surface binding, contributes to reduced Cr bioavailability in the nutrient solution. The application of biochar also increased the pH and decreased the electrical conductivity (EC) of the growth medium, thereby creating a more favorable rhizosphere environment. At the physiological level, biochar treatment suppressed reactive oxygen species (ROS) accumulation, decreased lipid peroxidation, and lowered membrane damage (assessed via relative electrolyte conductivity). Furthermore, antioxidant enzyme activities-including superoxide dismutase (SOD), peroxidase (POD), and catalase (CAT)-were significantly enhanced. These improvements contributed to better chloroplast integrity, enhanced sugar and protein synthesis, and reduced oxidative stress. Compared to untreated controls, biochar-treated plants exhibited significantly greater biomass, lower Cr accumulation, increased chlorophyll and nutrient contents, and elevated levels of soluble metabolites, collectively demonstrating biochar’s effectiveness in alleviating Cr toxicity and promoting overall plant health.

## Conclusion and future perspectives

In the current study chromium (Cr) contamination has been shown to adversely affect the growth, physiological processes, and biochemical responses of hydroponically grown Chinese cabbage (*Brassica rapa* L.), primarily by inducing oxidative stress, impairing photosynthetic efficiency, reducing nutrient uptake, and causing significant physiological damage. In this study, the application of biochar effectively alleviated these toxic effects by reducing Cr bioavailability, enhancing antioxidant enzyme activity, promoting chlorophyll synthesis, and improving nutrient absorption. Among the treatments, the highest biochar concentration (2.5 g/L, BC3) yielded the most notable improvements in plant biomass, chlorophyll content, and stress resilience. Mechanistic analyses using X-ray Photoelectron Spectroscopy (XPS) and Fourier Transform Infrared Spectroscopy (FTIR) confirmed the reduction of Cr (VI) to Cr (III) on the biochar surface, indicating that Cr detoxification was mediated through a combination of physical adsorption, chemical reduction via surface functional groups, and physiological regulation of stress-responsive pathways. These findings were synthesized into a three-level mechanistic model encompassing adsorption, physiological adjustment, and molecular signaling (Fig. 11). The broader implications of this research are threefold. First, in the context of global agricultural sustainability, biochar filtration offers a low-cost, scalable solution for mitigating heavy metal stress in hydroponic systems, particularly in water-scarce or industrially polluted regions, where nearly 20% of agricultural land is threatened by heavy metal contamination (FAO, 2022). Second, the use of invasive plant biomass for biochar production supports circular economy principles by simultaneously addressing resource recycling and environmental remediation, aligning with Sustainable Development Goal (SDG) 12. Third, the adsorption–physiological regulation mechanism demonstrated here is broadly applicable and can be adapted for the remediation of other heavy metals such as cadmium and lead, providing valuable insights for soilless agriculture, vertical farming, and urban food systems. In terms of practical application, the biochar filtration unit developed in this study can be integrated into hydroponic facilities for clean vegetable production and medicinal plant cultivation, and the optimal dosage identified (2.5 g/L) could be extended to pre-treatment systems for industrial effluents, including electroplating and tanning wastewater. Nevertheless, further research is needed to optimize biochar performance through feedstock selection, pyrolysis tuning, and functional modification. Long-term studies assessing biochar stability across multiple growth cycles and its interaction with soil microbiota and nutrient dynamics will be essential for broader agricultural deployment. Additionally, elucidating the molecular basis of biochar-mediated plant defense, particularly genes associated with antioxidant responses and metal transport, will further strengthen its role in sustainable heavy metal remediation strategies.

## Electronic supplementary material

Below is the link to the electronic supplementary material.

Supplementary Material 1

## Data Availability

Data is provided within the manuscript or supplementary information files.
