# Supplementary material for: Assessment of biochar filter application in improving chromium stress tolerance and plant physiology in Chinese cabbage (Brassica rapa) under a flow-through water setup
Source: BMC Biotechnol. 2025 Jul 19;25:74. doi: 10.1186/s12896-025-01010-3 (PMC12276666; doi:10.1186/s12896-025-01010-3)
Supplement: Supplementary file 1 — Supplementary Material 1 [file 12896_2025_1010_MOESM1_ESM.docx]

**Supplementary information**

**Assessment of biochar filter application in improving chromium stress tolerance and plant physiology in Chinese cabbage (Brassica rapa) under a flow-through water setup** Shuangqi Yue^1,2#^, Weidong Li^1,2#^, Fengyue Qin^1,2^, Menglu Dong^1,2^, Guojie Weng^1,2^, Hayssam M. Ali^3^, Jiechang Weng^4*^, Sajid Mehmood^1,2*^

^1^Center for Eco-Environment Restoration of Hainan Province, School of Ecology,

Hainan University, Haikou, 570228, China

^2^School of Topical Agriculture and Foresty, Hainan University, Haikou, 570228, China

^3^Department of Botany and Microbiology, College of Science, King Saud University, Riyadh– 11451, Saudi Arabia

^4^Hainan Provincial Ecological and Environmental Monitoring Center, Haikou, 570228, China.

#These authors contributed equally to this paper

***Corresponding authors**

**Dr.** **Jiechang Weng**; E-mail: [wjchang9033@126.com](mailto:wjchang9033@126.com)

**Dr. Sajid Mehmood**: E-mail: [drsajid@hainanu.edu.cn](about:blank)

**Section 2. BET surface analysis of biochar**

The surface area and pore structure of the biochar were characterized via nitrogen adsorption-desorption isotherms at 77 K, and the results are summarized in Table S2 and Figure S3. Parameters including BET surface area, total pore volume, and average pore diameter were analyzed to determine the structural suitability of the material for Cr adsorption. According to IUPAC classification, pores between 2 and 50 nm are considered mesopores [1–3]. The average pore diameter of the biochar was 4.8607 nm, placing it firmly in the mesoporous category. The isotherm observed (Figure S3b) corresponds to a type IV curve with an H4 hysteresis loop-typical of materials that contain both micropores and mesopores as well as narrow slit-like pores. This is consistent with the rough, porous morphology observed in SEM images and supports the dual pore structure of the material. The shape of the hysteresis loop and the continued nitrogen uptake at higher relative pressures suggest that multilayer adsorption and capillary condensation are occurring, indicative of mesoporosity with some contribution from micropores [4]. The desorption curve also showed a relatively narrow pore size distribution, with most pores falling in the range of 2.5-10 nm, further confirming the mesoporous structure. These structural features facilitate effective physical adsorption and pore-filling of Cr species. The BET results, as outlined in Table S2, confirm that the biochar has a high surface area and a well-defined mesoporous network, which enhance its adsorption capacity and make it an effective material for heavy metal remediation in aqueous environments.

**Section 1. XPS analysis of biochar**

X-ray Photoelectron Spectroscopy (XPS) was employed to investigate the surface chemistry and valence states of carbon and chromium on the biochar before and after Cr (VI) adsorption. XPS not only identifies elemental composition but also provides insights into valence state changes and surface redox processes involved during adsorption [4, 5]. As shown in Figure S4(a) and Table S3, the C1s spectra before and after adsorption exhibit characteristic peaks around 284.80 eV and 284.78 eV, which are attributed to C–H, C=C, and C–O bonds [6], consistent with FTIR results. These peaks correspond to surface functional groups such as hydroxyl and carbonyl groups, which are implicated in Cr binding. A reduction in carbon intensity and slight peak shifts after adsorption suggest the involvement of redox processes, where Cr (VI) may be partially reduced to Cr (III) by surface oxygen-containing groups, accompanied by oxidation of the carbon matrix. Additionally, evidence of coordination between Cr and carboxylic functional groups forming Cr–O–C bonds was observed, which may alter surface charge distribution and influence surrounding pH and EC values [7]. Table S3 shows that the full width at half maximum (FWHM) of the C1s peak decreased from 4.00 eV to 1.39 eV after adsorption, indicating increased homogeneity in carbon species, possibly due to selective coverage or modification of disordered carbon by Cr, as also visualized in Figure S4(a). The O1s peak shifted significantly from 537.70 eV to 529.30 eV (Δ = –8.4 eV), suggesting a transition from high-binding-energy oxygen species to low-binding-energy forms. This shift further supports the formation of Cr–O–Cr or Cr–O–C linkages, in agreement with FTIR signals observed at 1957.86 cm^-1^ and 1076.14 cm^-1^, confirming the interaction of Cr with C=O and C–O groups [8]. An increase in the O/C ratio from 0.29 to 0.35 was also noted, reflecting oxygen incorporation through Cr (VI) reduction and subsequent formation of Cr–O species, as well as surface oxidation via dissolved oxygen in water. The Cr 2p_3/2_ binding energy at 576.30 eV (Table S3, Figure S4b) lies between known values for Cr (III) (574-576 eV) and Cr (VI) (578-580 eV), indicating the coexistence of both species with Cr (III) as the dominant form. This is supported by EDS results, which detected surface-localized Cr (0.2%), and corroborated by a decrease in FTIR signal intensity for C=O/C–O groups. The XPS signal localization further confirms that chromium is mainly enriched at the biochar surface, consistent with micropore/mesopore interactions observed by SEM.

**References**

1. Schüth F, Schmidt W. Microporous and mesoporous materials. Adv Mater. 2002. https://doi.org/10.1002/1521-4095(20020503)14:9<629::AID-ADMA629>3.0.CO;2-B.

2. Zheng Y, Baudet BA, Delage P, Pereira JM, Sammonds P. Pore changes in an illitic clay during one-dimensional compression. Geotechnique. 2022. https://doi.org/10.1680/jgeot.21.00206.

3. Ding C, He J, Wu H, Zhang X. Nanometer pore structure characterization of taiyuan formation shale in the lin‐xing area based on nitrogen adsorption experiments. Minerals. 2021. https://doi.org/10.3390/min11030298.

4. Khalil TE, Khalil DA, Elbadawy HA, El-Sayed DS, El-dissouky A. An integrated experimental and computational study of modified biochar using biomass waste for the effective extraction of hazardous Cr(VI) from aqueous solutions. J Water Process Eng. 2025;74 March:107805.

5. Zou C, Xu Z, Nie F, Guan K, Li J. Application of hydroxyapatite-modified carbonized rice husk for the adsorption of Cr(VI) from aqueous solution. J Mol Liq. 2023. https://doi.org/10.1016/j.molliq.2022.121137.

6. Ray SK, Pant B, Park M, Bastakoti BP. Rice husk-derived sodium hydroxide activated hierarchical porous biochar as an efficient electrode material for supercapacitors. J Anal Appl Pyrolysis. 2023. https://doi.org/10.1016/j.jaap.2023.106207.

7. Yang Z, Wang J, Zhao N, Pang R, Zhao C, Deng Y, et al. A novel biochar-based 3D composite for ultrafast and selective Cr(VI) removal in electroplating wastewater. Biochar. 2024;6.

8. Li X, Wu Q, Chen D, Tamjidur RS, Fan P, Wang G, et al. Efficient chromium Cr (VI) removal from wastewater through modified cycad’s leaf biochar: insights into adsorption-reduction mechanisms and kinetic analysis. Biomass and Bioenergy. 2025;200 January:107994.


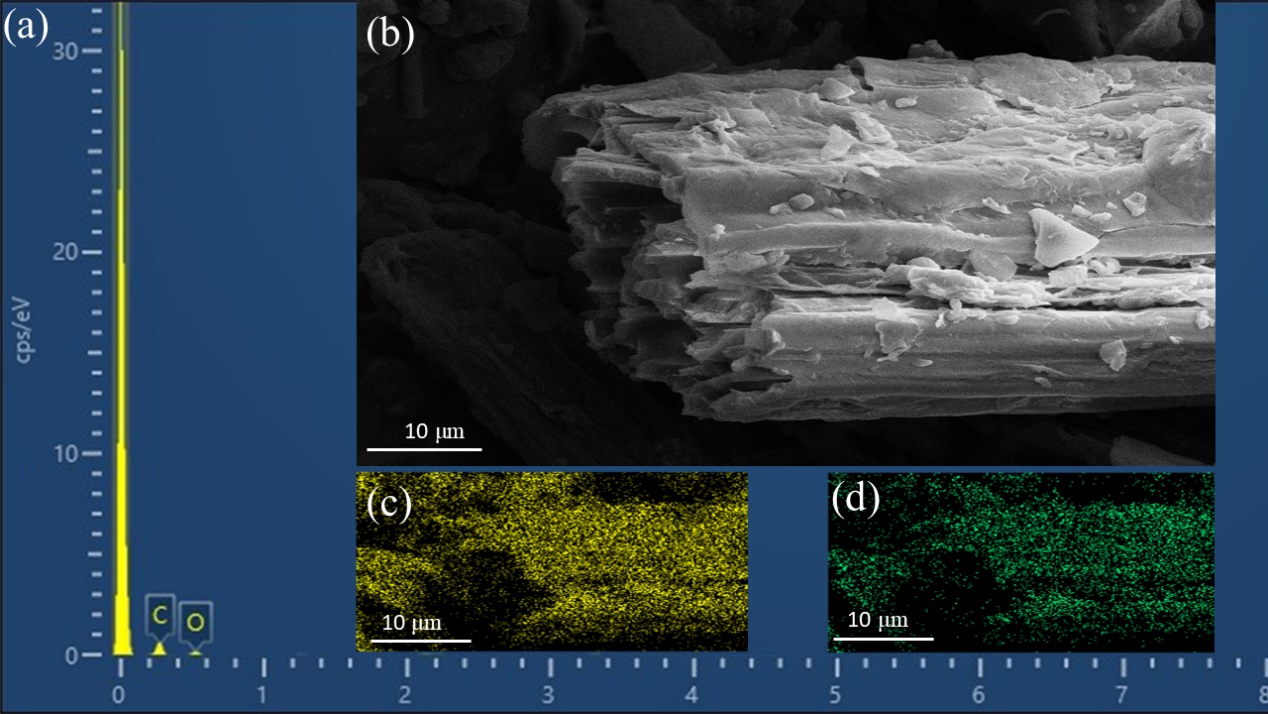


**Figure S1.** Energy-dispersive X-ray spectroscopy (EDS) and scanning electron microscopy (SEM) analysis of biochar before chromium adsorption. (a) EDS spectrum showing the elemental composition of biochar. (b) SEM image (c-d) Elemental mapping images, where (c) represents carbon distribution, (d) shows oxygen distribution.


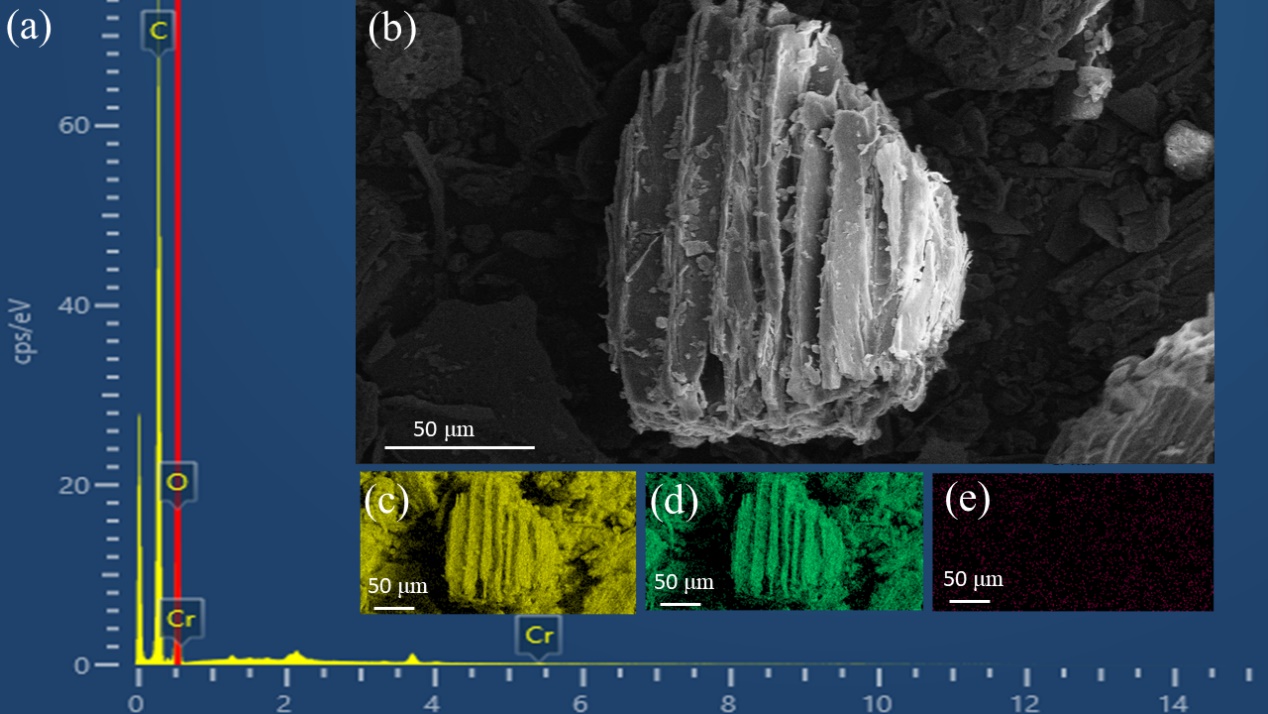


**Figure S2.** Energy-dispersive X-ray spectroscopy (EDS) and scanning electron microscopy (SEM) analysis of biochar after chromium adsorption. (a) EDS spectrum showing the elemental composition of biochar. (b) SEM image (c-e) Elemental mapping images, where (c) represents carbon distribution, (d) shows oxygen distribution, and (e) highlights chromium localization on the biochar surface.


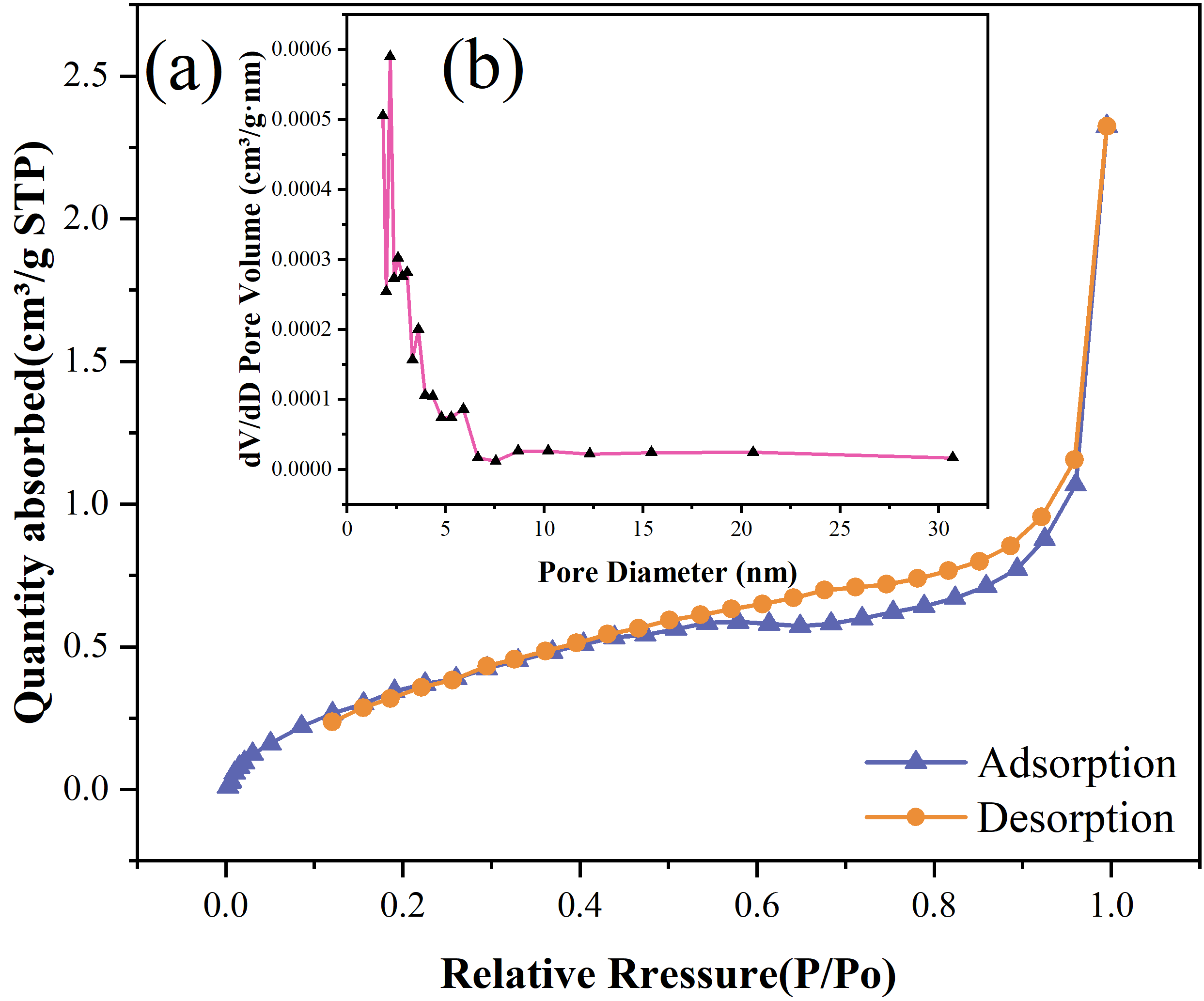


**Figure S3.** (a) Nitrogen adsorption-desorption isotherm of the biochar at 77 K (b) Barrett-Joyner-Halenda (BJH) pore size distribution curve derived from the desorption branch, highlighting a predominant pore diameter in the mesoporous range (2-5 nm).


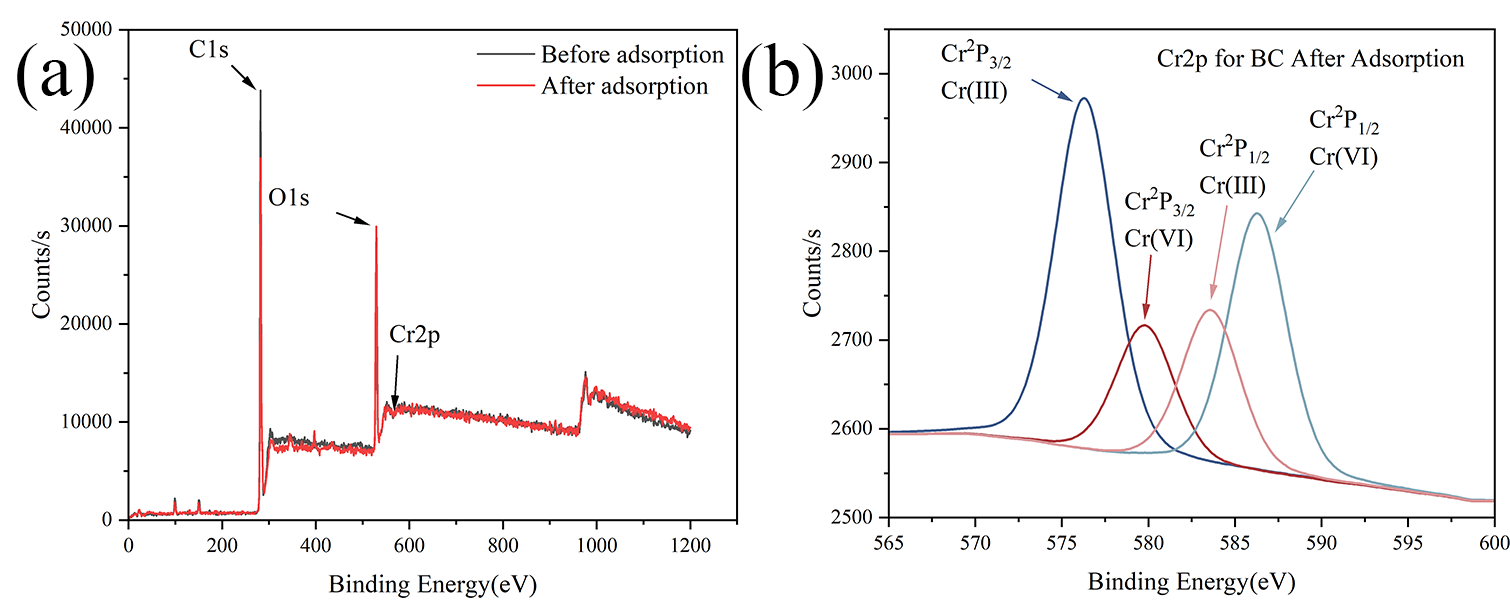


**Figure S4.** (a) XPS survey spectra of biochar before and after Cr (VI) adsorption. (b) High-resolution Cr2p spectrum of biochar after Cr (VI) adsorption.


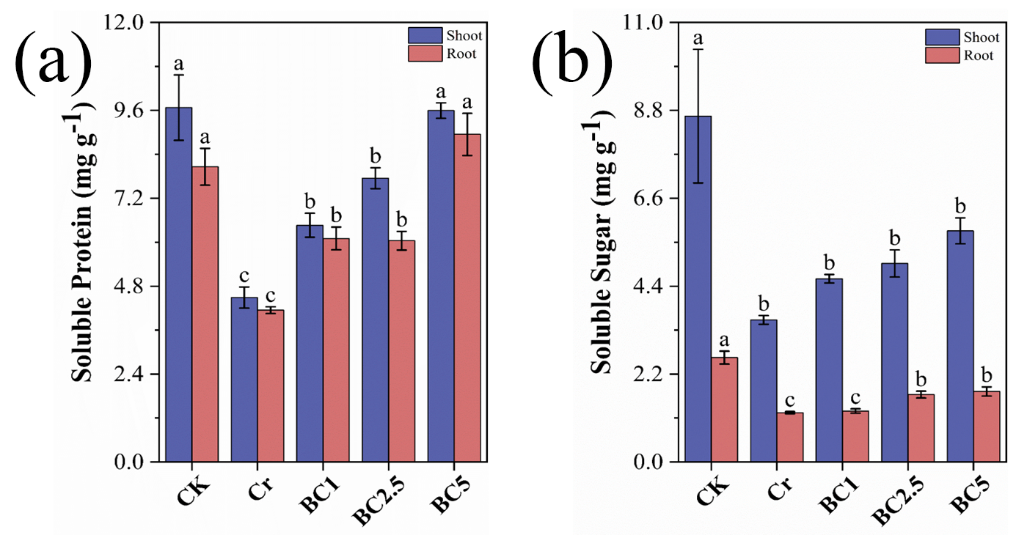


**Figure S5.** Effects of chromium stress and biochar treatment on (a) soluble protein, and (b) soluble sugar. Results are the mean values ± standard deviation (n = 3). Error bars indicate standard deviations. Different small letters on the bars indicate significant differences among treatments at P<0.05.


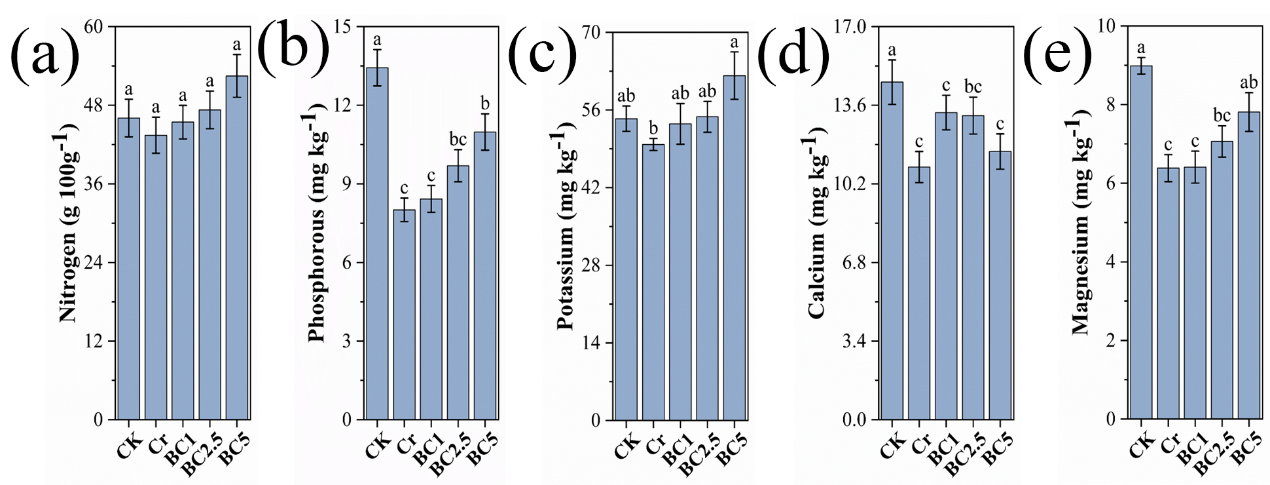


**Figure S6.** Effects of chromium stress and biochar treatment on (a) N content, (b) P content, (c) K content, (d) Ca content and (e) Mg content in Chinese cabbage tissues of different treatment group. Results are the mean values ± standard deviation (n = 3). Error bars indicate standard deviations. Different small letters on the bars indicate significant differences among treatments at P<0.05.

**Table S1.** EDS data of biochar element percentage in some regions before and after adsorption.

| Element | pre-adsorption (weight %) | post-adsorption (weight %) |
| --- | --- | --- |
| C | 68.2 | 66.3 |
| O | 31.6 | 33.4 |
| Cr | - | 0.2 |

**Table S2.** BET surface properties of biochar. The data were obtained from the nitrogen adsorption-desorption isotherms using the BET and BJH models.

| **BET Surface Area**  **(m^2^/g)** | **Pore Volume**  **(cm^3^/g)** | **Average Pore Size**  **(nm)** | **Pore type** |
| --- | --- | --- | --- |
| 1.5148 | 0.001357 | 4.8607 | Mesoporous |

**Table S3.** XPS analysis data of biochar before and after adsorption.

| **Name** | | **Peak BE**  **eV** | **FWHM eV** | **Atomic %** | **O/C-ratio** |
| --- | --- | --- | --- | --- | --- |
| Before adsorption | C1s | 284.80 | 4.00 | 77.10 | 0.29 |
|  | O1 | 537.70 | 3.36 | 22.40 |  |
| After adsorption | C1s | 284.78 | 1.39 | 68.23 | 0.35 |
|  | O1 | 529.30 | 2.65 | 24.17 |  |
|  | Cr2p3 | 576.30 | 3.98 | 0.14 |  |
